# Neuroprotective Effects of Myricetin on PTZ-Induced Seizures in Mice: Evaluation of Oxidation, Neuroinflammation and Metabolism, and Apoptosis in the Hippocampus

**DOI:** 10.3390/cimb46080527

**Published:** 2024-08-15

**Authors:** Grigory Demyashkin, Ekaterina Blinova, Migran Grigoryan, Mikhail Parshenkov, Polina Skovorodko, Vladimir Ius, Anastasia Lebed, Petr Shegay, Andrei Kaprin

**Affiliations:** 1 Laboratory of Histology and Immunohistochemistry, I.M. Sechenov First Moscow State Medical University (Sechenov University), Trubetskaya st., 8/2, 119048 Moscow, Russia; bev-sechenov@mail.ru (E.B.); migran1278@mail.ru (M.G.); skovorodko.polina2345@gmail.com (P.S.); ius.vova2017@yandex.ru (V.I.); ilebed.nastya22@gmail.com (A.L.); 2 Department of Digital Oncomorphology, National Medical Research Centre of Radiology, 2nd Botkinsky Pass., 3, 125284 Moscow, Russia; dr.shegai@mail.ru (P.S.); kaprin@mail.ru (A.K.); 3 Department of Urology and Operative Nephrology, Peoples’ Friendship University of Russia (RUDN University), Miklukho-Maklaya Str.6, 117198 Moscow, Russia

**Keywords:** epilepsy, NeuN, caspase-8, pentyltetrazole, apoptosis, hippocampus, neurons

## Abstract

Epilepsy is one of the most frequently diagnosed neurological diseases, but the neurobiological basis of the disease remains poorly understood. Immunophenotyping CBA mice brain (NeuN and caspase-8) in parallel with hippocampal neurons’ functional status and survival rate assessment during acute epileptic PTZ-induced seizures is of particular interest. The aims of this study were to investigate the involvement of NeuN and caspase-8 in cell cycle regulation and the death of hippocampal neurons during PTZ-induced seizures in mice and to assess the therapeutic efficacy of Myricetin in the aforementioned experimental settings. Male CBA mice (*n* = 340) were divided into six groups to investigate the neuroprotective and antiepileptic effects of Myricetin and Valproic Acid in the PTZ-induced seizure model. Group I (control, *n* = 20) received a single intraperitoneal injection of NaCl 0.9% solution. Group II (PTZ only, *n* = 110) received a single intraperitoneal 45 mg/kg PTZ to induce seizures. Group III (Myricetin + PTZ, *n* = 90) was administered Myricetin orally at 200 mg/kg for 5 days, followed by a PTZ injection. Group IV (Valproic Acid + PTZ, *n* = 80) received intraperitoneal Valproic Acid at 100 mg/kg for 5 days, followed by PTZ. Group V (Myricetin + NaCl, *n* = 20) received Myricetin and NaCl. Group VI (Valproic Acid + NaCl, *n* = 20) received Valproic Acid and NaCl. Seizure severity was monitored using the modified Racine scale. Behavioral assessments included sensorimotor function tests, motor coordination using the rotarod test, and cognitive function via the Morris water maze. Brain tissues were collected and analyzed for oxidative stress markers, including malondialdehyde (MDA), superoxide dismutase (SOD), and glutathione (GSH). Blood samples were analyzed for cytokine levels (IL-1β, IL-6, and TNF-α). Histological studies involved H&E and Nissl staining to evaluate general histopathology and neuronal density. Immunohistochemical analysis was conducted using antibodies against NeuN and caspase-8 to assess neuronal cell cycle regulation and apoptosis. PTZ-induced seizures caused significant oxidative stress and inflammation, leading to neuronal damage. Biochemical analyses showed elevated levels of MDA, SOD, GSH, IL-1β, IL-6, and TNF-α. Histological and immunohistochemical evaluations revealed a significant increase in caspase-8-positive neurons and a decrease in NeuN-positive neurons in the hippocampus and other brain regions, correlating with seizure severity. Myricetin and Valproic Acid treatments reduced oxidative stress markers and neuronal damage. Both treatments resulted in moderate neuronal protection, with fewer damaged neurons observed in the hippocampus, dentate gyrus, and other brain areas compared to the PTZ-only group. Summarizing, Myricetin administration showed promising neuroprotective effects. It significantly reduced oxidative stress markers, including MDA, and restored antioxidant enzyme activities (SOD and GSH), suggesting its antioxidative potential. Myricetin also effectively attenuated the elevation of pro-inflammatory cytokines IL-1β, IL-6, and TNF-α, indicating strong anti-inflammatory properties. Behavioral assessments revealed that Myricetin improved cognitive and motor functions in PTZ-treated mice, with notable reductions in seizure severity and mortality rates. Histological analyses supported these behavioral findings, with Nissl staining showing reduced neuronal damage and NeuN staining indicating better preservation of neuronal integrity in Myricetin-treated groups. Additionally, caspase-8 staining suggested a significant reduction in neuronal apoptosis.

## 1. Introduction

Today, epilepsy is one of the most frequently diagnosed neurological diseases, affecting approximately 2% of the population [1,2]. The most severe manifestation of epilepsy is status epilepticus (epistatus), which is characterized by prolonged generalized epileptic seizures associated with the progression of cognitive dysfunction against the background of dystrophic changes in hippocampal structures. 

Despite more than several centuries of research into epileptogenesis and the development of treatment protocols, the neurobiological basis of the disease remains poorly understood. It is well known that patients with epilepsy have fewer hippocampal neurons and gliosis: mesial temporal sclerosis (hippocampal sclerosis). The International League Against Epilepsy (ILAE) distinguishes three forms of hippocampal sclerosis: typical (type I) and atypical (type II and III) [3,4]. Nevertheless, the role of mesial temporal sclerosis in epileptogen esis is still widely debated, as the causal relationship with seizures has not been established.

How only one seizure can be able to start neurological changes, and how can these be considered as an initial factor able to start the epileptogenesis process? The study of disturbances in the cell cycle of neurons, their differentiation, and death are considered relevant for neurodegenerative diseases, particularly neurodegeneration.

One of the most used methods for studying epilepsies is modeling in experiments since the pathomorphological changes that occur in the hippocampus of animals, primarily a decrease in the number of neurons, are like those in patients. The administration of pentyltetrazole (PTZ) is a popular rodent model of epilepsy, causing a subconvulsive chemical stimulus leading to a progressive increase in convulsive activity, reaching a climax with systematic seizures [5,6,7]. Cognitive and emotional issues appear in rodent behavior disorders due to damage predominantly to the dorsal hippocampus neurons. Other brain regions, such as the thalamus, fimbriae, and amygdala, are also important during and after seizure initiation.

For morphofunctional assessment of the state of neural tissues, a neuron-specific neuronal nuclear antigen (NeuN, Neuronal Nuclei) was used. NeuN is a widely used neuron-specific marker that allows the identification and quantification of mature neurons in the hippocampus and other parts of the brain, both normally and in various neurodegenerative diseases. NeuN is associated with secretory granules, small vesicles, or cytosol elements in neurons [8,9]. Alterations in various metabolic and molecular processes in the hippocampus are of great interest. It is known that the hippocampus is vulnerable to some neurological diseases, the etiopathogenesis of which is associated with impaired functional status of neurons, as well as altered levels of antioxidant enzymes in the hippocampus. It is possible that changes in cell morphology and the distribution of NeuN-immunoreactive neurons in different subregions of the hippocampus will help to unravel the mechanisms of seizures. The quantitative neuronal changes by NeuN-analysis may be manifested by cognitive impairments as they are hippocampus-dependent tasks in the PTZ-associated seizure model.

It is known that in most brain structures, cell death activation occurs through the caspase cascade, where caspases-8,-9,-10 act as the initiator and caspases-3,-6,-7 act as terminal molecules [10,11,12,13,14,15,16]. Nevertheless, the available data are insufficient to provide a complete picture of the nature of the caspase cascade in PTZ-induced seizures. Currently, in PTZ-induced seizures, the role of the receptor pathway of apoptosis, which triggers caspase 8 after the interaction of Fas and FasL receptors, has not been sufficiently studied. In a few studies, it was shown that after an epileptic seizure, there is an increase in the level of caspase-3 and caspase-8, which does not exclude a correlation between the activation of the caspase cascade and neuronal death [5,17]. However, the mechanism of intraneuronal regulation of the extrinsic apoptosis pathway was unclear. Uncovering this regulatory mechanism will help to understand the origin of seizures and key events of epileptogenesis. Moreover, the question remains open: “How seizures and caspase-8 are involved in neuroinflammation and oxidative stress?”.

Currently, a significant concern for scientists from various specialties, including pharmacologists, morphologists, and neurologists, is the search for affordable and high-quality drugs for the treatment of epilepsy. Special attention is being paid to phytopreparations due to their potential protective and antiepileptic properties. Unlike chemically synthesized drugs, they have a less pronounced cytotoxic effect on nervous tissue. These substances are capable of not only reducing the degree of neuroinflammation and oxidative stress but also enhancing metabolic processes in neurons, such as the synthesis of gamma-aminobutyric acid (GABA). Their antihypoxic and anticonvulsant effects aim to decrease the excitability and seizure readiness of the motor areas of the brain, thus suppressing seizures without pronounced side effects. Therefore, research is currently focused on investigating the protective and anticonvulsant properties of plant-derived substances, which may open new possibilities for seizure control and for the effective and safe treatment of epilepsy.

Myricetin, 3,3′,4′,4,5,5′,7-hexahydroxyflavone, is a flavonoid extracted from various parts of the Chinese myrtle (Myrica rubra) and other fruits and vegetables [18,19]. Myricetin possesses several biologically active properties, including antioxidative, antitumor, nephroprotective, and anti-inflammatory effects. Only one experimental study has demonstrated the antiepileptic effect of Myricetin on brain neurons [20]. The authors of this study focused primarily on the chronic phase/kindling of the PTZ-induced model. However, the morphofunctional states of neurons, their metabolism, and cell death in the acute phase of PTZ-induced seizures and the background of Myricetin administration remain unexplored, as well as its neuroantioxidant properties.

Based on the neuroprotective and anticonvulsant properties of Myricetin, confirmed by its ability to regulate the BDNF-TrkB signaling pathway and modulate GABA receptor activity [20], we hypothesized that Myricetin administration increases the synthesis of various biological substances, thereby enhancing GABAergic neurotransmission. Myricetin administration may have a direct effect on both PTZ-induced seizures and an indirect effect on oxidative stress while reducing neural tissue damage to the hippocampus and other brain regions. Our study aims to reveal the mechanisms of neuronal functional status abnormalities in the acute phase of PTZ-induced seizures, which theoretically may open new therapeutic opportunities for the treatment of epilepsy.

## 2. Research Objective

The aim of this study is twofold. 

### Research Tasks

1. First, to test the hypothesis that NeuN and caspase-8 are involved in the cell cycle regulation of hippocampal neurons and their death induced by systemic seizures, we performed an immunohistochemical study of the brains of mice subjected to PTZ-induced seizures.
2. Second, we aimed to test the hypothesis that Myricetin administration increases the synthesis of neurosteroids and to assess the effects of Myricetin on early induced PTZ seizures, focusing on its potential to mitigate oxidative stress and neuropathological damage in the hippocampus. These effects will be evaluated using histological and molecular biological methods.

## 3. Materials and Methods

### 3.1. Animal Model for In Vivo Study

Male sexually mature mice of the CBA population (30–35 g; 12 weeks old; *n* = 340) were obtained from the animal facility and housed under standard laboratory conditions (temperature of 22 ± 2 °C; humidity of 50–60%; 12 h light/dark cycle). Mice had access to a standard pellet diet and water ad libitum and were allowed to acclimate to the laboratory conditions for 5–7 days prior to the experiments. All efforts were made to minimize animal suffering and to reduce the number of animals used.

The animals were allocated into six groups:

Group I (*n* = 20): control/intact. Single intraperitoneal injection of NaCl 0.9% solution.

Group II (*n* = 110): PTZ only. Mice received a single intraperitoneal injection of PTZ at a dosage of 45 mg/kg to induce seizures without prior administration of Myricetin or Valproic Acid.

Group III (*n* = 90): Myricetin + PTZ. Mice received oral administration of Myricetin at a dose of 200 mg/kg for 5 days, followed by a single intraperitoneal injection of pentylenetetrazole (PTZ) at a dosage of 45 mg/kg to induce seizures.

Group IV (*n* = 80): Valproic Acid + PTZ. Mice received intraperitoneal injections of Valproic Acid at a dosage of 100 mg/kg for 5 days, followed by a single intraperitoneal injection of PTZ at a dosage of 45 mg/kg to induce seizures.

Group V (*n* = 20): Myricetin + NaCl. Mice received oral administration of Myricetin at a dose of 200 mg/kg for 5 days, followed by a single intraperitoneal injection of NaCl 0.9% solution.

Group VI (*n* = 20): Valproic Acid + NaCl. Mice received intraperitoneal injections of Valproic Acid at a dosage of 100 mg/kg for 5 days, followed by a single intraperitoneal injection of NaCl 0.9% solution.

Myricetin, a flavonoid extracted from various parts of the Chinese myrtle (Myrica rubra), was administered at a dosage of 200 mg/kg orally to assess its neuroprotective and antiepileptic effects. Myricetin treatment was carried out for 5 days prior to the induction of seizures with PTZ. The dose of Myricetin (200 mg/kg) was selected experimentally based on a pilot study, where several doses of Myricetin (50, 100, 200, 500 mg/kg) were tested. The Valproic Acid + PTZ group and Valproic Acid + NaCl group received intraperitoneal injections of Valproic Acid at a dosage of 100 mg/kg for 5 days prior to a single PTZ or NaCl administration.

To assess the severity of the PTZ-induced seizures, the experimental animals were monitored for 30 min after the seizure, and the modified Racine scale [21] was used, as shown in Table 1.

Animals of all groups were withdrawn from the experiment at these points after behavioral tests were performed. Each time point involved euthanizing animals using a combination of ketamine (100 mg/2 mL) at 300 mg/kg and xylazine 2% (20 mg/1 mL) at 30 mg/kg, administered intraperitoneally.

### 3.2. Experimental Design

Before starting the experiment, baseline behavioral tests were conducted in all groups to establish a seizure-free baseline. After a 5-day treatment period with either Myricetin or Valproic Acid, behavioral tests were again performed to assess any treatment-induced changes. Mice were then injected once intraperitoneally with either PTZ at a dose of 45 mg/kg to induce seizures or 0.9% NaCl solution for the control groups.

The severity of the PTZ-induced seizures was assessed by monitoring the experimental animals for 40 min after the seizure using the modified Racine scale (Table 1). Seizure types were categorized and tracked over a 40 min period post-injection, including myoclonic seizures characterized by whole-body twitching, clonic seizures identified by rhythmic jerking motions often accompanied by stupor or abnormal posturing, and tonic seizures, which involved sustained muscle contractions leading to hindlimb extension. The observation protocol allowed for a comprehensive assessment of the seizure phases and severity, providing critical data on the anticonvulsant efficacy of the treatments administered. During the study, we paid attention to any mild seizures or behavioral changes in the animals after a 15 min observation period. Additionally, the duration of each observed seizure was measured, as changes in seizure duration depend on their severity. An important criterion was the delay to the first seizure after PTZ injection. Monitoring the frequency, duration, and latency of seizures was crucial for subsequent molecular studies. 

**Figure 1:**
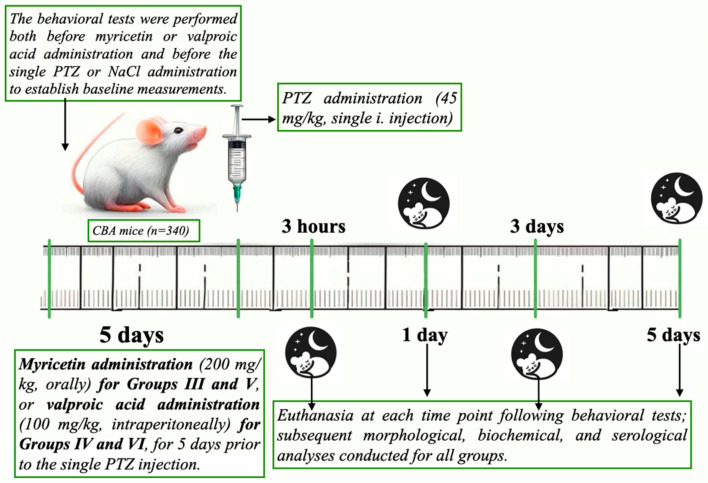
Study design for PTZ-induced seizures groups and Myricetin administration; III—Myricetin + PTZ; IV—Valproic Acid + PTZ; V—Myricetin + NaCl; VI—Valproic Acid + NaCl.

The obtained brain preparations were examined for markers of oxidative stress, and biochemical blood tests were performed to further analyze the effects of PTZ, Myricetin, and Valproic Acid treatment. The experimental design is illustrated in Figure 1. Behavioral assessments were conducted at four specific time points: 3 h, 1 day, 3 days, and 5 days post-PTZ-injection. At each time point, a subset of animals from each group was euthanized for subsequent analysis. 

### 3.3. Behavioral Tests

All behavioral evaluations were conducted under the same conditions. Behavioral tests were performed both before Myricetin administration and before PTZ administration to establish baseline measurements. 

### 3.4. Sensorimotor Functions

Sensorimotor functions were evaluated using the foreign object removal test. For this purpose, two squares of adhesive tape (5 mm^2^) were glued to the palmar surface of the upper limbs, and the subsequent behavior of the animal was evaluated [22].

### 3.5. Motor Coordination

Motor coordination was assessed using the rotarod test. The time taken for the mouse to fall from the rotating rod at a speed of 20 rotations per minute was recorded. The test was conducted for 3 min [23].

### 3.6. Motor Functions

Total distance traveled was assessed in the open-field test during a 3 min session [24].

### 3.7. Cognitive Impairment

**Figure 2:**
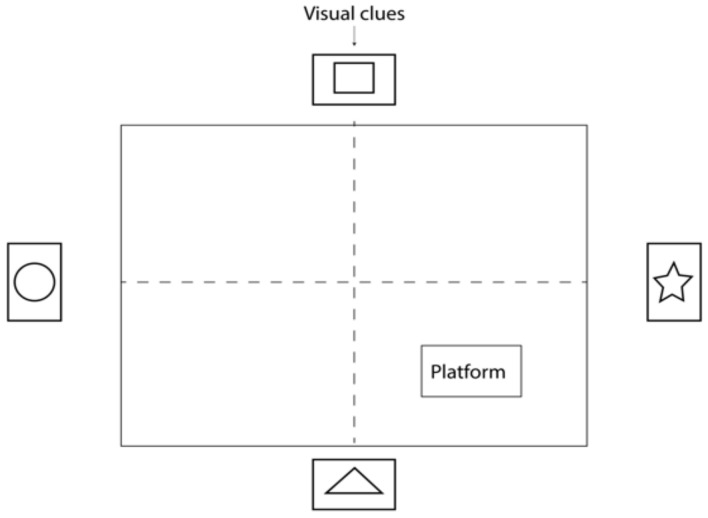
Morris water maze test.

Cognitive functions were assessed using the Morris water maze. The pool was a square cavity conventionally divided into 4 sectors and filled with water. The side of the square was approximately 1.4 m. To conceal the platform, the water in the pool was tinted, and visual cues were placed against each wall (Figure 2). During the training period of 4 days before the experiment, each animal was placed in the water 10 cm away from the pool wall in a defined pattern, and the time to find the platform was recorded. Each animal was placed in the pool four times for no more than 1 min with a 30 s break between trials. If the rodent did not find the platform within 1 min, it was placed on the platform and left for 15 s, after which training continued according to the same scheme [25]. After PTZ administration and before sacrifice, animals were subjected to a control test in which the platform was removed from the sector. The time taken to find the target sector and the time spent in the target sector were assessed. Time performance was measured manually by an independent observer.

### 3.8. Oxidative Stress Markers Evaluation

Brain homogenates were prepared by homogenizing 0.1 g of brain tissue in 4.5 mL of cold potassium buffer (pH 7.4). The solution was then centrifuged at 13,000 rpm for 5 min at 4 °C. The supernatant was collected and stored at −80 °C. Levels of malondialdehyde (MDA), a biomarker of lipid peroxidation, superoxide dismutase (SOD), and glutathione (GSH) in the brain homogenates were measured using ELISA kits (Lifespan Biosciences, Seattle, WA, USA).

### 3.9. Biochemical Blood Test

Serum levels were determined in the blood of animals after they were euthanized. These levels were measured using commercial ELISA cytokines IL-1β, IL-6 (Bender MedSystems, Vienna, Austria), and TNF-α (Assaypro, St Charles, MO, USA) kits according to the manufacturer’s instructions.

### 3.10. Histological Study

Brain fragments were fixed in 10% neutral buffered formalin (pH 7.2–7.4) and embedded in paraffin according to standard techniques. After deparaffinization and rehydration, paraffin sections (3 μm thick) were stained with hematoxylin and eosin (H&E) to assess general histopathology. For a more detailed analysis of neuronal cell loss, additional sections were stained with Nissl stain to visualize neuronal cell bodies and assess neuronal density and integrity. Quantitative analysis of cell loss in the hippocampus was performed using Nissl-stained sections to identify cell damage in the brain after PTZ-induced seizures.

### 3.11. Immunohistochemical Study

Immunohistochemical study was performed according to the standard protocol in manual mode. Citrate buffer was used for antigen retrieval, pH 6.0, 10×. Primary mouse monoclonal antibody to NeuN/Neuronal Marker (1B7, 1:100; Abcam, Waltham, MA, USA) and polyclonal rabbit antibody to Caspase 8 (CAP4; 1:50; Abcam, Waltham, MA, USA) were used according to the protocol of manufacturer. Secondary—universal antibodies (anti-mouse or anti-rabbit IgG; HiDef Detection™ HRP Polymer system, “Cell Marque”, Rocklin, CA, USA). DAB was used as a chromogen. No staining could be revealed when the primary antibodies were omitted. Microscopic analysis was performed using a video microscopy system (Leica DM2000 microscope, Leica Microsystems, Wetzlar, Germany; Leica ICC50 HD camera, Leica Microsystems, Wetzlar, Germany) and Image J (Version 1.51) and Leica Application Suite (LAS) Version 4.9.0 programs.

The number of living (bright pink) or damaged (dark) neurons was counted on Hematoxylin-and-Eosin- (H&E) and Nissl-stained sections. The neuronal density for each animal was defined as the average number of neurons per optical field, calculated from four sections. Specifically, the mean number of cells was counted from eight optical fields in the CA1, CA3, and CA4 regions, and from twelve optical fields in the dentate gyrus (DG) for each mouse.

Quantitative analysis of NeuN-positive cells in a 0.02 mm^2^ section was used to evaluate the immunohistochemical reaction, and the results were presented as arithmetic mean (M) and standard deviation (SD). The number of caspase-8-positive cells was counted at a magnification of ×400, in 10 fields of view. To assess the proportion of living or damaged NeuN- and caspase-8-positive cells, the number of such cells was counted using sections adjacent to H&E and Nissl-stained sections.

The neuronal density (N) per 1 mm^2^ was calculated using the following formula:

$$N\;in\;1\;{mm}^{2}=\frac{\sum N\;\times \;1,000,000\;{mkm}^{2}}{N\;optical\;fields\;\times \;S\;one\;field\;\left({mkm}^{2}\right)}$$

where the following apply:

- ∑N is the total number of neurons counted;
- N is the number of optical fields analyzed;
- S is the area of one optical field.

To ensure the accuracy of the data obtained, several independent opinions were obtained: independent scientists, who did not know the experimental conditions, quantified cells in the hippocampus using projections of images on a screen.

### 3.12. Statistical Analysis

All data are presented as the mean ± standard deviation. The obtained data were processed using the SPSS for Windows statistical software package (version 10.0; IBM Corp., Armonk, NY, USA). A one-way analysis of variance (ANOVA) followed by Duncan’s Multiple Range Test was used for multiple group comparisons. In the analysis of specific parameters, such as the number of dark cells in various hippocampal areas during the recovery period, a two-way ANOVA was employed to reveal differences in the vulnerabilities of the studied regions, followed by Dunnett’s post hoc test for comparison of the experimental and control groups. An alpha level of 0.05 was used as the significance criterion in all the tests. The data are given as the mean ± standard error of the mean. Differences were considered statistically significant at *p* < 0.05.

## 4. Results

### 4.1. PTZ-Induced Seizures

Prior to the initiation of the experimental protocol, baseline behavioral assessments were conducted across all groups. During this period, no seizure activity was observed, corresponding to a score of −1 on the Racine scale. This baseline was maintained throughout the initial phase of the study, including the five-day pre-treatment period with either Myricetin or Valproic Acid. The absence of PTZ administration during this phase ensured that no convulsive episodes were recorded, establishing a consistent and seizure-free baseline for subsequent evaluations.

In the I, V, and VI groups, no seizure activity was recorded throughout the duration of the study. This absence of convulsive behavior remained consistent with the baseline assessments, which were measured at −1 on the Racine scale. These groups were subjected to the same behavioral tests at all designated time points, and their responses consistently reflected the initial baseline state. The lack of PTZ administration ensured that these control groups did not exhibit any epileptiform activity, thereby serving as a reliable baseline for comparison against the treatment and seizure-induced groups. 

**Figure 3:**
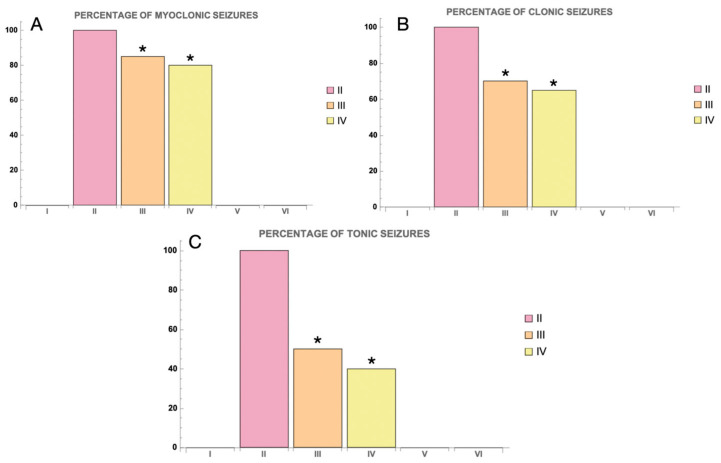
The percentage of animals presenting with myoclonic, clonic, and tonic seizures induced by PTZ in different groups. Legend: (**A**)—percentage of myoclonic seizures; (**B**)—percentage of clonic seizures; (**C**)—percentage of tonic seizures. Groups are numbered according to the study design. Statistically significant differences are indicated by symbols: *—comparison with Group II (PTZ only) (*p * < 0.05). Groups: I—intact; II—PTZ only; III—Myricetin + PTZ; IV—Valproic Acid + PTZ; V—Myricetin + NaCl; VI—Valproic Acid + NaCl.

PTZ administration successfully induced seizures in all animals in the group where only PTZ was used (Group II). Animals in this group exhibited 100% seizure activity, and a marked mortality was observed (Figure 3). Specifically, 60% of the animals in Group II died from PTZ-induced convulsions. In the Myricetin + PTZ group (Group III), seizure activity was significantly reduced compared to the group receiving PTZ alone. The frequency of myoclonic, clonic, and tonic seizures was 85%, 70%, and 50%, respectively, and mortality was reduced to 25% (Figure 3). This indicates a marked protective effect of Myricetin against PTZ-induced seizures. In the Valproic Acid + PTZ group (Group IV), an even greater reduction in seizure activity and mortality was observed. The frequency of myoclonic, clonic, and tonic seizures in this group was 80%, 65%, and 40%, respectively, and mortality was only 20%, highlighting the efficacy of Valproic Acid in reducing seizure severity (Figure 3). Only surviving animals from all experimental groups were included in further studies.

### 4.2. Latency to Seizure Onset

**Figure 4:**
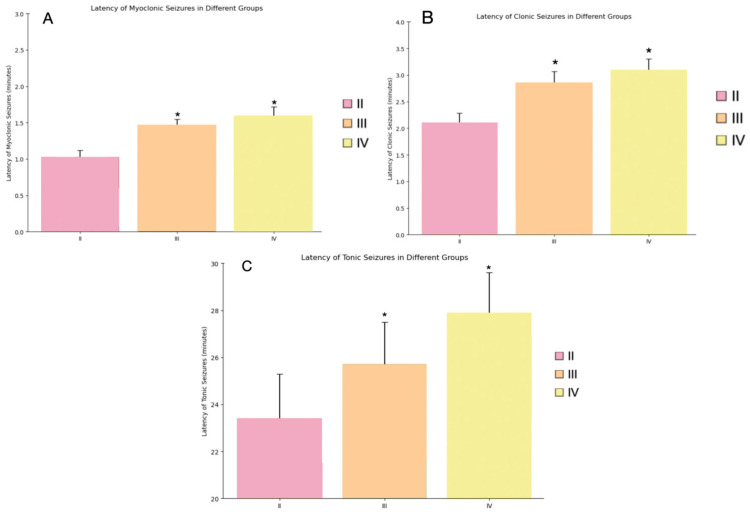
The latency of different types of seizures (in minutes) induced by PTZ in different groups. Legend: (**A**)—latency of myoclonic seizures (for II, III and IV—groups); (**B**)—latency of clonic seizures (for II, III and IV—groups); (**C**)—latency of tonic seizures (for II, III and IV—groups). Groups are numbered according to the study design. The bars represent mean ± SD. Statistically significant differences are indicated by symbols: *—comparison with II group (PTZ only) (*p * < 0.05). Groups: II—PTZ only; III—Myricetin + PTZ; IV—Valproic Acid + PTZ.

The latency to seizure onset was significantly delayed in both the Myricetin + PTZ (Group III) and Valproic Acid + PTZ (Group IV) groups compared to the PTZ-only group (Group II) (Figure 4). In Group II, myoclonic seizures began at 1.02 ± 0.1 min, clonic seizures began at 2.10 ± 0.18 min, and tonic seizures began at 23.4 ± 1.90 min (Figure 4). In contrast, Group III exhibited myoclonic seizures at 1.45 ± 0.10 min, clonic seizures at 2.85 ± 0.22 min, and tonic seizures at 25.7 ± 1.80 min (Figure 4). Group IV showed the most significant delay, with myoclonic seizures at 1.60 ± 0.12 min, clonic seizures at 3.10 ± 0.20 min, and tonic seizures at 27.9 ± 1.70 min (Figure 4). The differences in latency to seizure onset were statistically significant (*p* < 0.05) when comparing Group II to Groups III and IV.

### 4.3. Seizure Duration and Severity

**Figure 5:**
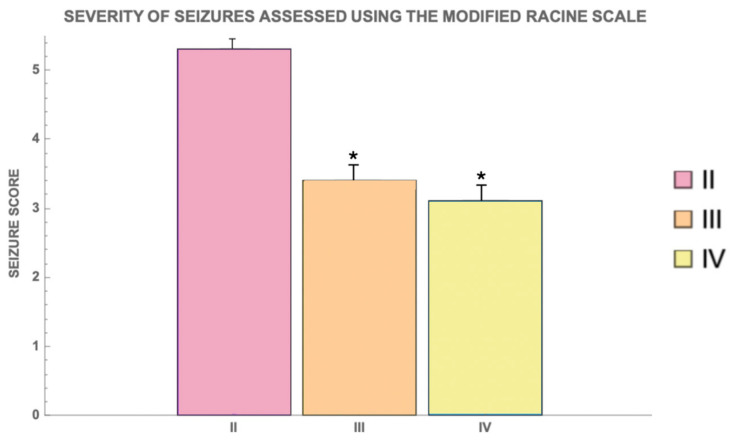
Severity of seizures assessed using the modified Racine scale for different groups. Groups are numbered according to the study design. The score for the remaining groups not represented in the graph is −1 (no seizures were recorded). The bars represent mean ± SD. Statistically significant differences are indicated by symbols: *—comparison with II group (PTZ only) (*p * < 0.05). II—PTZ only; III—Myricetin + PTZ; IV—Valproic Acid + PTZ.

The duration and severity of seizures were assessed using the modified Racine scale (Figure 5). Group II animals had the highest seizure severity scores, with an average score of 5.6 ± 0.3, indicating frequent occurrences of tonic–clonic seizures and wild jumping. In contrast, Group III and Group IV exhibited average scores of 3.4 ± 0.2 and 3.1 ± 0.2, respectively, showing a significant reduction in the severity of seizures (*p* < 0.05 for both comparisons with Group II) (Figure 5).

### 4.4. Mortality Rate

**Figure 6:**
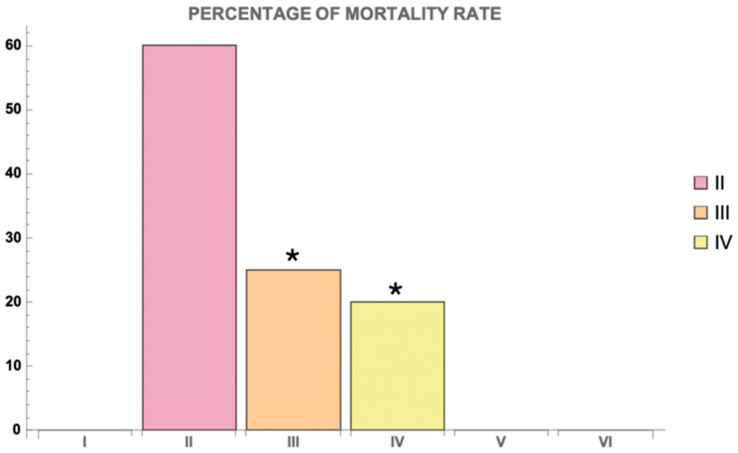
Percentage of mortality rate for different groups. Groups are numbered according to the study design. Statistically significant differences are indicated by symbols: *—comparison with Group II (PTZ only) (*p * < 0.05). Groups: I—intact; II—PTZ only; III—Myricetin + PTZ; IV—Valproic Acid + PTZ; V—Myricetin + NaCl; VI—Valproic Acid + NaCl.

The mortality rate in Group II was 60%, indicating a high lethality associated with PTZ-induced seizures (Figure 6). In contrast, the mortality rates in Groups III and IV were significantly lower, at 25% and 20%, respectively (*p* < 0.05) (Figure 6). This significant reduction in mortality suggests a protective effect of both Myricetin and Valproic Acid against PTZ-induced lethality.

The following number of animals was included in the further study to ensure uniform distribution within the experimental groups, according to the recommendations for preclinical studies, and to simplify the statistical processing of data: Group I (*n* = 20); Group II (*n* = 40); Group III (*n* = 60); Group IV (*n* = 60); Group V (*n* = 20); Group VI (*n* = 20).

### 4.5. Evaluation of Behavioral Tests

**Figure 7:**
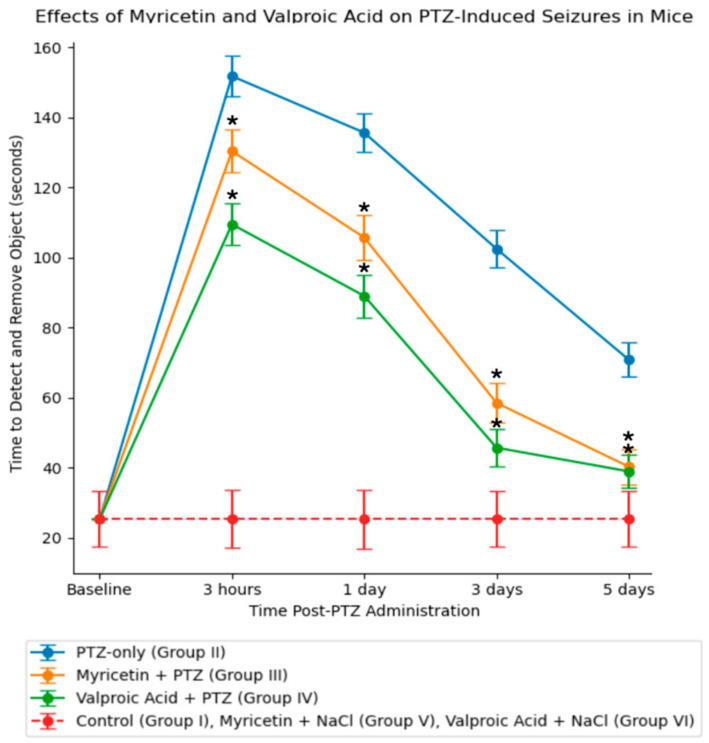
Adhesive removal test for different groups. Data represent mean ± SD. Statistically significant differences are indicated by symbols: *—comparison with group II (PTZ only) (*p * < 0.05). Groups: I—intact; II—PTZ only; III—Myricetin + PTZ; IV—Valproic Acid + PTZ; V—Myricetin + NaCl; VI—Valproic Acid + NaCl.

Foreign object removal. The foreign object-removal test evaluated the effects of Myricetin and Valproic Acid on PTZ-induced seizures in mice, measured in seconds. Baseline assessments showed consistent performance in the control (Group I), Myricetin + NaCl (Group V), and Valproic Acid + NaCl (Group VI) groups, with an average time of 25.29 ± 8.19 s (Figure 7). In the PTZ-only group (Group II), the time to detect and remove the object significantly increased to 151.75 ± 5.76 s three hours post-PTZ (*p* < 0.05). Times decreased to 135.62 ± 5.45 s on day 1, 102.45 ± 5.23 s on day 3, and 70.78 ± 4.90 s by day 5 (Figure 7). The Myricetin + PTZ group (Group III) showed reduced impairment, with times of 130.34 ± 6.12 s at three hours (*p* < 0.05 compared to Group II), 105.67 ± 6.45 s on day 1, 58.45 ± 5.67 s on day 3, and 40.23 ± 5.12 s by day 5 (Figure 7). The Valproic Acid + PTZ group (Group IV) also demonstrated improvement, with times of 109.45 ± 5.98 s at three hours (*p* < 0.05 compared to Group II), 89 ± 6.12 s on day 1, 45.67 ± 5.45 s on day 3, and 38.90 ± 4.78 s by day 5 (Figure 7). These results indicate that both Myricetin and Valproic Acid significantly mitigate PTZ-induced motor and cognitive impairments.

**Figure 8:**
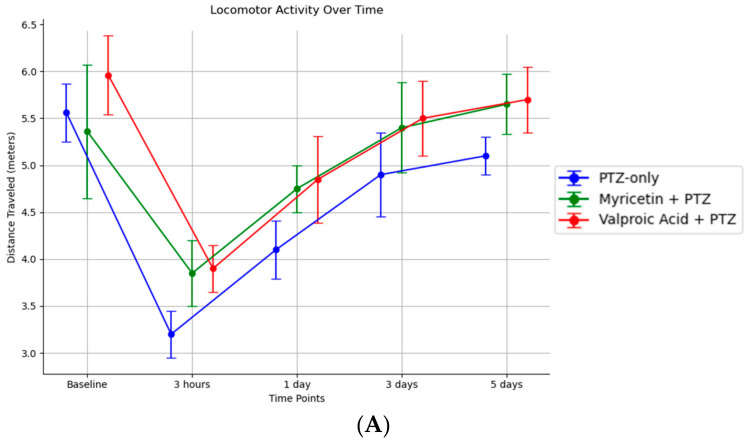

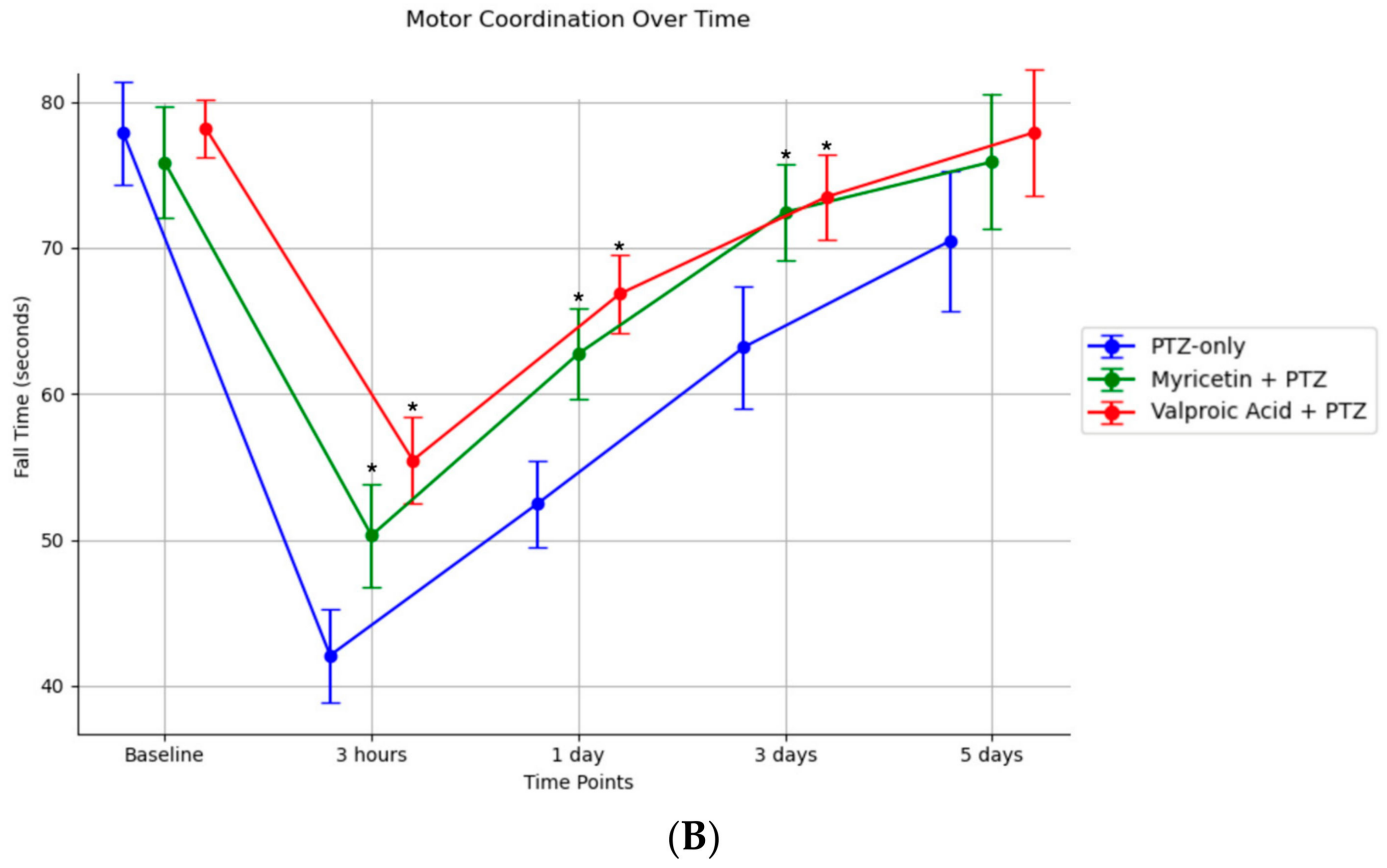
(**A**) Motor activity test results for different groups. Data represent mean ± SD. Groups: I—intact; II—PTZ only; III—Myricetin + PTZ; IV—Valproic Acid + PTZ; V—Myricetin + NaCl; VI—Valproic Acid + NaCl. (**B**) Motor coordination test results for different groups. Data represent mean ± SD. Statistically significant differences are indicated by symbols: *—comparison with group II (PTZ only) (*p * < 0.05). Groups: I—intact; II—PTZ only; III—Myricetin + PTZ; IV—Valproic Acid + PTZ; V—Myricetin + NaCl; VI—Valproic Acid + NaCl.

Motor functions. Baseline locomotor activity was consistent across all groups, with an average total distance traveled of approximately 5.56 m in 3 min. Three hours after PTZ administration, the PTZ-only group (Group II) showed a marked decrease in locomotor activity, averaging 3.20 m. Activity gradually recovered over the subsequent days, reaching 4.10 m at 1 day, 4.90 m at 3 days, and 5.10 m at 5 days (Figure 8A). The Myricetin + PTZ group (Group III) displayed a faster recovery, with activity recorded at 3.85 m at 3 h, 4.75 m at 1 day, 5.40 m at 3 days, and 5.65 m at 5 days (Figure 8A). The Valproic Acid + PTZ group (Group IV) also showed significant improvement, with 3.90 m at 3 h, 4.85 m at 1 day, 5.50 m at 3 days, and 5.70 m at 5 days (Figure 8A). Both Myricetin and Valproic Acid enhanced the recovery of locomotor activity following PTZ-induced impairment, as indicated by the improved distances traveled over time compared to the PTZ-only group.

Motor coordination. The rotating platform test was used to assess motor coordination. The baseline performance was similar in all groups, with a mean fall time of about 77.87 s. Three hours after PTZ administration, motor coordination decreased significantly in the group where only PTZ was used (Group II), with a mean fall time of 42.08 s. During the experiment, animals in this group showed a gradual improvement in motor coordination, with fall times of 52.45 s on day 1, 63.20 s on day 3, and 70.50 s on day 5 (Figure 8B).

The Myricetin + PTZ group (Group III) showed faster recovery: 50.30 s at 3 h (*p* < 0.05 compared to Group II), 61.75 s at 1 day (*p* < 0.05 compared to Group II), 71.45 s at 3 days (*p* < 0.05 compared to Group II), and 75.60 s at 5 days (Figure 8B). Similarly, the Valproic Acid + PTZ group (Group IV) showed significant improvement, with recovery times of 55.45 s after 3 h (*p* < 0.05 compared to Group II), 65.85 s after 1 day (*p* < 0.05 compared to Group II), 73.50 s after 3 days (*p* < 0.05 compared to Group II), and 76.90 s after 5 days (Figure 8B). By day 5, motor coordination in the Myricetin + PTZ and Valproic Acid + PTZ groups had fully recovered to baseline, indicating significant recovery from PTZ-induced impairment.

Spatial memory and cognitive function. Training was performed in all groups of animals before the beginning of the experiment without dividing them into subgroups. On the last day of training, the measured time was recorded as a baseline. Further comparisons were made between the PTZ-only, Myricetin + PTZ, Valproic Acid + PTZ, Myricetin + NaCl, and Valproic Acid + NaCl groups. At 3 h after PTZ administration, the time spent in the target quadrant was significantly shorter in the PTZ-only group compared to the control (baseline) group. Significant differences were also observed at 1 day and 3 days post-PTZ in the Myricetin + PTZ and Valproic Acid + PTZ groups compared to the PTZ-only group. No statistically significant differences were found at the other time points (Table 2).

**Figure 9:**
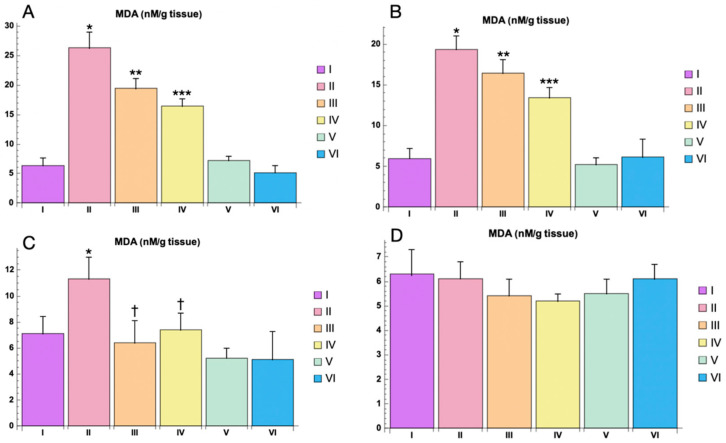
MDA levels in animal brain homogenate for different study groups: (**A**)—3 h, (**B**)—1 day, (**C**)—3 days, (**D**)—5 days. Experimental groups are numbered according to the study design. Data are presented as mean ±SD. Statistically significant differences compared to the control group are indicated by—* (*p * < 0.05); statistically significant differences between III and II are indicated by ** (*p * < 0.01); statistically significant differences between IV and II groups are indicated by *** (*p * < 0.001), statistically significant differences at 3 days between groups (II and III) with PTZ only is indicated by—† (*p * < 0.05). Groups: I—intact; II—PTZ only; III—Myricetin + PTZ; IV—Valproic Acid + PTZ; V—Myricetin + NaCl; VI—Valproic Acid + NaCl.

Oxidative stress markers evaluation. The levels of malondialdehyde (MDA), a key biomarker of lipid peroxidation, were significantly elevated in the PTZ-only group compared to the control group (*p * < 0.05). This increase in MDA levels indicates heightened oxidative stress due to PTZ-induced seizures. The administration of Myricetin (200 mg/kg) and Valproic Acid (100 mg/kg) led to a notable reduction in MDA levels (Figure 9).

The most pronounced changes were observed at 3 h post-PTZ administration. Myricetin significantly reduced MDA levels compared to the PTZ-only group (*p * < 0.01), while Valproic Acid showed an even greater reduction (*p* < 0.001). At 1 day post-PTZ, both Myricetin and Valproic Acid continued to show significant reductions in MDA levels compared to the PTZ-only group (*p * < 0.01 and *p * < 0.001, respectively). By 3 days post-PTZ, MDA levels in both the Myricetin and Valproic Acid groups were still significantly lower than those in the PTZ-only group (*p * < 0.05). By 5 days post-PTZ, no statistically significant differences in MDA levels were observed between any of the groups, indicating a return to baseline oxidative stress levels (*p >* 0.05) (Figure 9).

In the control groups receiving either Myricetin or Valproic Acid in combination with NaCl 0.9% (Groups V and VI), there were no statistically significant changes (*p >* 0.05) in MDA levels compared to the baseline control group, demonstrating that these treatments alone did not alter oxidative stress markers (Figure 9).

**Figure 10:**
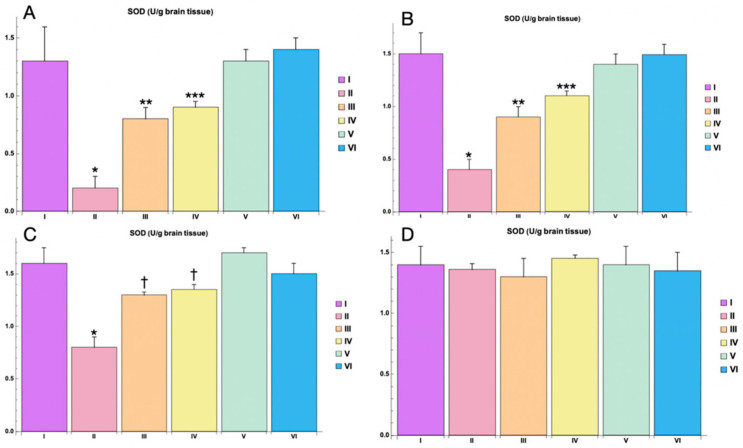
SOD levels in animal brain homogenate for different study groups: (**A**)—3 h, (**B**)—1 day, (**C**)—3 days, (**D**)—5 days. Experimental groups are numbered according to the study design. Data are presented as mean ±SD. Statistically significant differences compared to the control group are indicated by—* (*p * < 0.05); statistically significant differences between III and II are indicated by ** (*p * < 0.01); statistically significant differences between IV and II groups are indicated by *** (*p * < 0.001), statistically significant differences at 3 days between groups (II and III) with PTZ only is indicated by—† (*p * < 0.05). Groups: I—intact; II—PTZ only; III—Myricetin + PTZ; IV—Valproic Acid + PTZ; V—Myricetin + NaCl; VI—Valproic Acid + NaCl.

The activity of superoxide dismutase (SOD), an important antioxidant enzyme, was significantly decreased in the PTZ-only group compared to the control group (*p * < 0.05) at 3 h post-PTZ administration. This reduction in SOD activity highlights the oxidative stress induced by PTZ (Figure 10). At 3 h post-PTZ, the administration of Myricetin and Valproic Acid mitigated the decrease in SOD activity. Specifically, Myricetin significantly attenuated the reduction in SOD levels compared to the PTZ-only group (*p * < 0.01), while Valproic Acid demonstrated an even more pronounced effect (*p * < 0.001) (Figure 10). At 1 day post-PTZ administration, SOD activity in the Myricetin and Valproic Acid groups continued to show significant improvement compared to the PTZ-only group. The levels of SOD in the Myricetin group remained significantly higher (*p * < 0.01), and the Valproic Acid group maintained a highly significant elevation in SOD activity (*p * < 0.001) (Figure 10). By 3 days post-PTZ, SOD activity in both the Myricetin and Valproic Acid groups was still significantly higher than that in the PTZ-only group, indicating a recovery trend in antioxidant defense (*p * < 0.05 for both groups). By 5 days post-PTZ, no statistically significant differences in SOD activity were observed between any of the groups, indicating a normalization of oxidative stress markers (Figure 10).

In the control groups receiving either Myricetin or Valproic Acid in combination with NaCl 0.9% (Groups V and VI), there were no statistically significant changes in SOD activity compared to the baseline control group. This demonstrates that the treatments alone did not adversely affect antioxidant enzyme levels.

**Figure 11:**
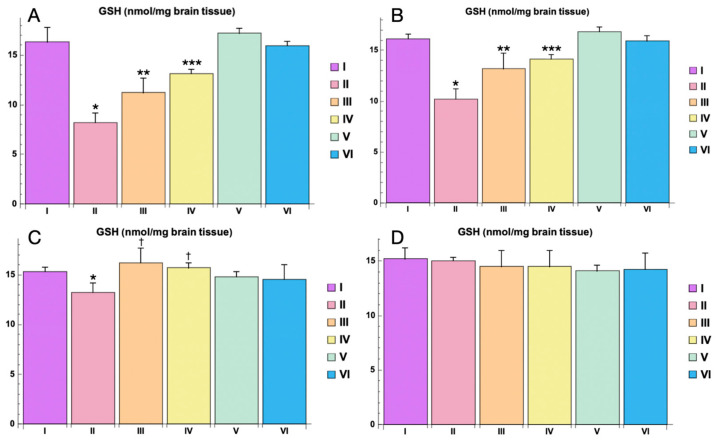
GSH levels in animal brain homogenate for different study groups: (**A**)—3 h, (**B**)—1 day, (**C**)—3 days, (**D**)—5 days. Experimental groups are numbered according to the study design. Data are presented as mean ± SD. Statistically significant differences compared to the control group are indicated by—* (*p * < 0.05); statistically significant differences between III and II are indicated by ** (*p * < 0.01); statistically significant differences between IV and II groups are indicated by *** (*p * < 0.001), statistically significant differences at 3 days between groups (II and III) with PTZ only is indicated by—† (*p * < 0.05). Groups: I—intact; II—PTZ only; III—Myricetin + PTZ; IV—Valproic Acid + PTZ; V—Myricetin + NaCl; VI—Valproic Acid + NaCl.

The levels of glutathione (GSH) were significantly decreased in the PTZ-only group compared to the control group (*p * < 0.05) at 3 h post-PTZ administration. This reduction in GSH levels underscores the oxidative stress burden induced by PTZ (Figure 11). At 3 h post-PTZ, the administration of Myricetin and Valproic Acid ameliorated the decrease in GSH levels. Myricetin significantly mitigated the reduction in GSH compared to the PTZ-only group (*p * < 0.01), while Valproic Acid exhibited a more substantial effect (*p * < 0.001) (Figure 11). At 1 day post-PTZ administration, GSH levels in the Myricetin and Valproic Acid groups continued to show significant improvement compared to the PTZ-only group. GSH levels in the Myricetin group were significantly higher (*p * < 0.01), and the Valproic Acid group maintained a highly significant elevation in GSH levels (*p * < 0.001) (Figure 11). By 3 days post-PTZ, GSH levels in both the Myricetin and Valproic Acid groups were comparable to those in the control group, indicating a return to baseline antioxidant capacity (*p >* 0.05 for both groups) (Figure 11). At 5 days post-PTZ, GSH levels remained like control levels in all groups, indicating a full recovery of antioxidant capacity with no statistically significant differences observed (Figure 11). In the control groups receiving either Myricetin or Valproic Acid in combination with NaCl 0.9% (Groups V and VI), there were no statistically significant changes in GSH levels compared to the baseline control group. This confirms that the treatments alone did not negatively impact antioxidant levels.

### 4.6. Biochemical Blood Test

Blood biochemical analysis was performed to assess the levels of proinflammatory cytokines IL-1β, IL-6, and TNF-α in the blood serum of experimental animals at different time points after PTZ-induced seizures and treatment with Myricetin and Valproic Acid. We examined cytokine levels at all time points in the present study: before the start of the experiment (baseline); before PTZ treatment (after 5 days of drug treatment); and then at 3 h, 1 day, 3 days, and 5 days after seizure induction.

Before starting the experiment, we also measured the baseline blood levels of all cytokines to determine the initial values for accurate comparison throughout the study. This step was critical to establish a reference point and ensure that any changes observed in cytokine levels after treatment could be accurately attributed to the experimental interventions. By comparing these baseline values with post-treatment levels, we aimed to gain a clearer picture of the effects of administered compounds on the inflammatory response in the animal model.

**Figure 12:**
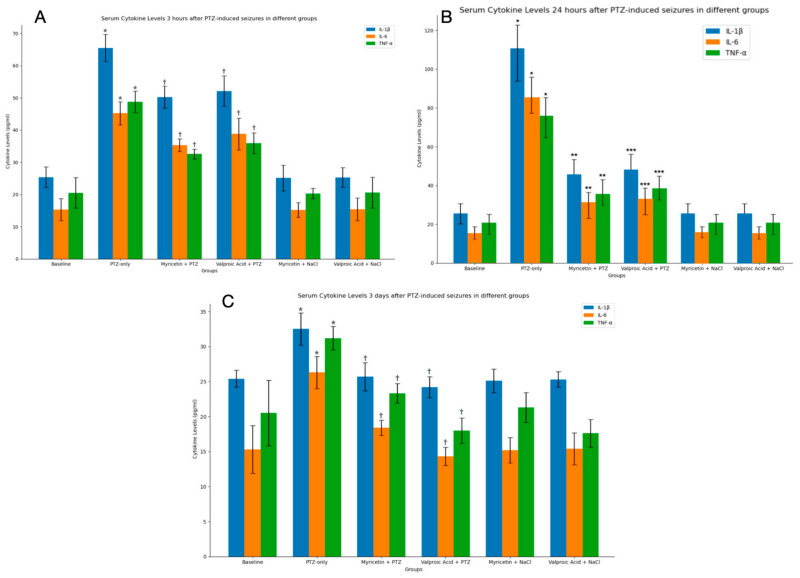
The cytokine levels after PTZ-induced seizures for different groups: (**A**)—after 3 h; (**B**)—after 24 h; (**C**)—after 3 days. Experimental groups are named according to the study design. Data are presented as mean ± SD. Statistically significant differences compared to the control group are indicated by—* (*p * < 0.05); statistically significant differences between III and II are indicated by ** (*p * < 0.01), and † (*p * < 0.05); statistically significant differences between IV and II groups are indicated by *** (*p * < 0.01), and † (*p * < 0.05). Groups: I—intact; II—PTZ only; III—Myricetin + PTZ; IV—Valproic Acid + PTZ; V—Myricetin + NaCl; VI—Valproic Acid + NaCl.

After 3 h after PTZ-induced seizures, serum levels of IL-1β, IL-6, and TNF-α were elevated in the PTZ-only group (Group II) compared to the control group (Group I), although the increase was less pronounced compared to the 24 h mark. Specifically, IL-1β levels increased to 65.5 ± 8.2 pg/mL from the baseline level of 25.4 ± 5.2 pg/mL (*p * < 0.05), IL-6 levels rose to 45.2 ± 7.6 pg/mL from 15.3 ± 3.4 pg/mL (*p * < 0.05), and TNF-α levels surged to 48.8 ± 7.3 pg/mL from 20.5 ± 4.7 pg/mL (*p * < 0.05) (Figure 12A).

In the Myricetin + PTZ group (Group III), the increase in cytokine levels was moderately attenuated compared to the PTZ-only group. IL-1β levels were 50.2 ± 6.4 pg/mL (*p * < 0.05 compared to Group II), IL-6 levels were 35.3 ± 6.0 pg/mL (*p * < 0.05), and TNF-α levels were 32.6 ± 5.5 pg/mL (*p * < 0.05). Similarly, in the Valproic Acid + PTZ group (Group IV), cytokine levels were also moderately reduced. IL-1β levels were 52.1 ± 6.7 pg/mL (*p * < 0.05 compared to Group II), IL-6 levels were 38.8 ± 5.9 pg/mL (*p * < 0.05), and TNF-α levels were 35.9 ± 6.2 pg/mL (*p * < 0.05) (Figure 12A).

For the control groups that received either Myricetin or Valproic Acid without PTZ induction (Groups V and VI), the levels of IL-1β, IL-6, and TNF-α were not significantly different from the control group (Group I), indicating that Myricetin and Valproic Acid alone do not affect the baseline levels of these cytokines (Figure 12A).

After 24 h, the serum levels of IL-1β, IL-6, and TNF-α were significantly elevated in the PTZ-only group (Group II) compared to the control group (Group I). Specifically, IL-1β levels increased to 110.5 ± 12.3 pg/mL from the baseline level of 25.4 ± 5.2 pg/mL (*p * < 0.05), IL-6 levels rose to 85.2 ± 10.8 pg/mL from 15.3 ± 3.4 pg/mL (*p * < 0.05), and TNF-α levels surged to 75.8 ± 9.6 pg/mL from 20.5 ± 4.7 pg/mL (*p * < 0.05) (Figure 12B).

In the Myricetin + PTZ group (Group III), the increase in cytokine levels was significantly attenuated compared to the PTZ-only group. IL-1β levels were 45.7 ± 7.8 pg/mL (*p* < 0.01 compared to Group II), IL-6 levels were 30.4 ± 6.1 pg/mL (*p * < 0.01), and TNF-α levels were 35.6 ± 7.4 pg/mL (*p * < 0.01). Similarly, in the Valproic Acid + PTZ group (Group IV), cytokine levels were also significantly reduced. IL-1β levels were 48.2 ± 8.0 pg/mL (*p * < 0.01 compared to Group II), IL-6 levels were 32.8 ± 5.9 pg/mL (*p * < 0.01), and TNF-α levels were 38.1 ± 6.8 pg/mL (*p * < 0.01) (Figure 12B).

For the control groups that received either Myricetin or Valproic Acid without PTZ induction (Groups V and VI), the levels of IL-1β, IL-6, and TNF-α were not significantly different from the control group (Group I), indicating that Myricetin and Valproic Acid alone do not affect the baseline levels of these cytokines. These results suggest that Myricetin and Valproic Acid significantly mitigate the PTZ-induced increase in pro-inflammatory cytokines in a comparable manner, highlighting their potential anti-inflammatory effects in the context of PTZ-induced seizures (Figure 12B).

After 3 days, serum levels of IL-1β, IL-6, and TNF-α in the PTZ-only group (Group II) compared to the control group (Group I) showed that cytokine levels started to normalize, being closer to baseline levels. Specifically, IL-1β levels were 32.5 ± 6.3 pg/mL, IL-6 levels were 27.3 ± 5.8 pg/mL, and TNF-α levels were 30.2 ± 5.5 pg/mL (*p * < 0.05 for all) (Figure 12C).

In the Myricetin + PTZ group (Group III), cytokine levels were very close to the control levels. IL-1β levels were 28.7 ± 5.2 pg/mL (*p >* 0.05 compared to Group I, *p * < 0.05 compared to Group II), IL-6 levels were 22.4 ± 4.9 pg/mL (*p >* 0.05 compared to Group I, *p * < 0.05 compared to Group II), and TNF-α levels were 24.3 ± 5.1 pg/mL (*p >* 0.05 compared to Group I, *p * < 0.05 compared to Group II). Similarly, in the Valproic Acid + PTZ group (Group IV), cytokine levels were also near control levels. IL-1β levels were 29.2 ± 5.5 pg/mL (*p >* 0.05 compared to Group I, *p * < 0.05 compared to Group II), IL-6 levels were 23.6 ± 5.2 pg/mL (*p >* 0.05 compared to Group I, *p * < 0.05 compared to Group II), and TNF-α levels were 25.0 ± 5.3 pg/mL (*p >* 0.05 compared to Group I, *p * < 0.05 compared to Group II) (Figure 12C).

For the control groups that received either Myricetin or Valproic Acid without PTZ induction (Groups V and VI), the levels of IL-1β, IL-6, and TNF-α were not significantly different from the control group (Group I), indicating that Myricetin and Valproic Acid alone do not affect the baseline levels of these cytokines. These results suggest that by 3 days post-PTZ induction, the levels of pro-inflammatory cytokines in the treated groups have largely normalized, highlighting the potential anti-inflammatory effects of Myricetin and Valproic Acid in the context of PTZ-induced seizures (Figure 12C).

By 5 days post-PTZ administration, the serum levels of IL-1β, IL-6, and TNF-α had normalized across all experimental groups. There were no statistically significant differences in cytokine levels between the PTZ-only group, the Myricetin + PTZ group, the Valproic Acid + PTZ group, and the control groups. This indicates a return to baseline levels of inflammatory markers, suggesting that the inflammatory response induced by PTZ was transient and resolved by the fifth day.

### 4.7. Morphological Study

**Figure 13:**
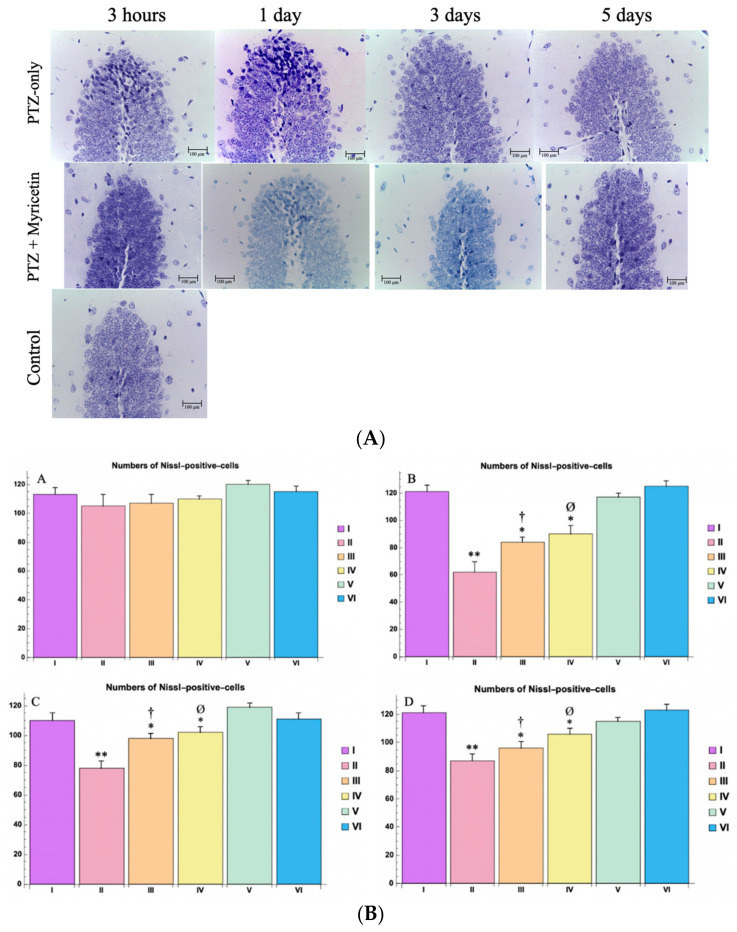
(**A**). Morphological picture of the hippocampus (dentate gyrus)at different time points of the experiment for different study groups.Nissl staining, magn,X400, Disintegration of “dark” neurons with signsofpycnosis, without demarcation between cytoplasm and nucleus. (**B**). The numbers of Nissl-positive-cells of dentate gyrus (the hippocampus zone) at different time points, per mm^2^: A—3 h, B—1 day, C—3 days, D—5 days. Experimental groups are numbered according to the study design. Data are presented as mean ±SD. Statistically significant differences compared to the control group are indicated by—* (*p * < 0.05), ** (*p * < 0.01); statistically significant differences between III and II are indicated by † (*p * < 0.01); statistically significant differences between IV and II groups are indicated by ø (*p * < 0.001). Groups: I—intact; II—PTZ only; III—Myricetin + PTZ; IV—Valproic Acid + PTZ; V—Myricetin + NaCl; VI—Valproic Acid + NaCl.

In the PTZ-only group, significant morphological changes were observed in the hippocampus after 24 h (after PTZ injections), particularly in the CA1, CA3, and CA4 fields, as well as in the dentate gyrus. Histological analysis revealed a moderate but statistically significant decrease in the number of cells in these areas (*p * < 0.05 for CA1, CA3, and CA4; *p * < 0.01 for the dentate gyrus). This decrease in cell number was associated with a significant increase in the number of damaged neurons in fields CA1, CA4, and the dentate gyrus (*p * < 0.05). A histochemical examination of hippocampal slides stained with Nissl at 3 h post-PTZ injection did not reveal any histoarchitectonic disturbances. However, 24 h after PTZ injection, an increase in the number of “dark” neurons with signs of pycnosis and hyperchromic staining was observed, indicating irreversible cell damage. On the 3rd and 5th day after PTZ administration, there was a tendency to decrease the number of pathological neurons. Cells with a characteristic damage profile stained positively for caspase 8. The cell borders of some hippocampal neurons in the CA3, dentate gyrus, and hilus regions were less clear, but the pycnotic nuclei were not visible (Figure 13A,B). PTZ induction also observed an increase in the number of “dark” neurons in the thalamic and hypothalamic nuclei, as well as motor structures such as the caudate nucleus and substantia nigra.

The morphological changes in the Myricetin + PTZ group were less pronounced compared to the group receiving PTZ alone. Further, 24 h after PTZ administration on the background of Myricetin, the number of neurons in the fields CA1, CA3, CA4, and dentate gyrus was close to control values, and the number of “dark” neurons was single. A similar pattern was observed with Nissl staining. This trend was maintained on 3 days and on 5 days after PTZ on the Myricetin background, indicating its protective effect (Figure 13A), as well as in the thalamic and hypothalamic nuclei and motor structures such as the caudate nucleus and substantia nigra.

The comparison drug (Valproic Acid) showed the best histological and histochemical picture at all terms of the study (Figure 13B).

In the control group (group I) and in the groups receiving Myricetin or Valproic Acid combined with NaCl 0.9% (groups V and VI), the histological picture and cellular composition of the hippocampus remained unchanged.

**Figure 14:**
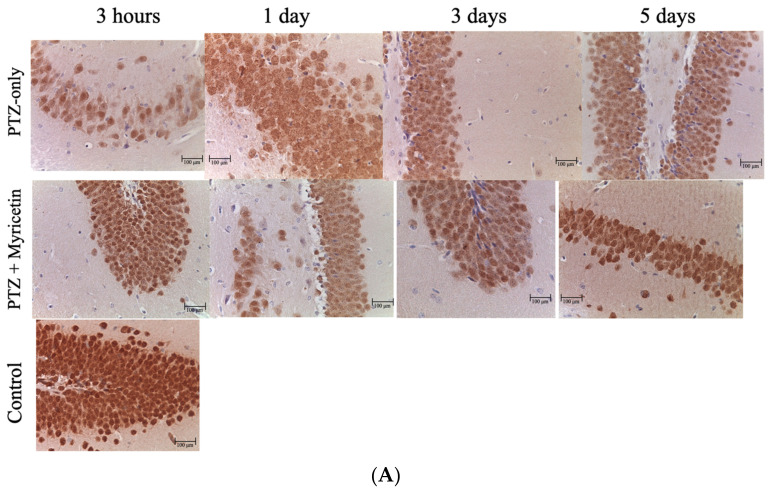

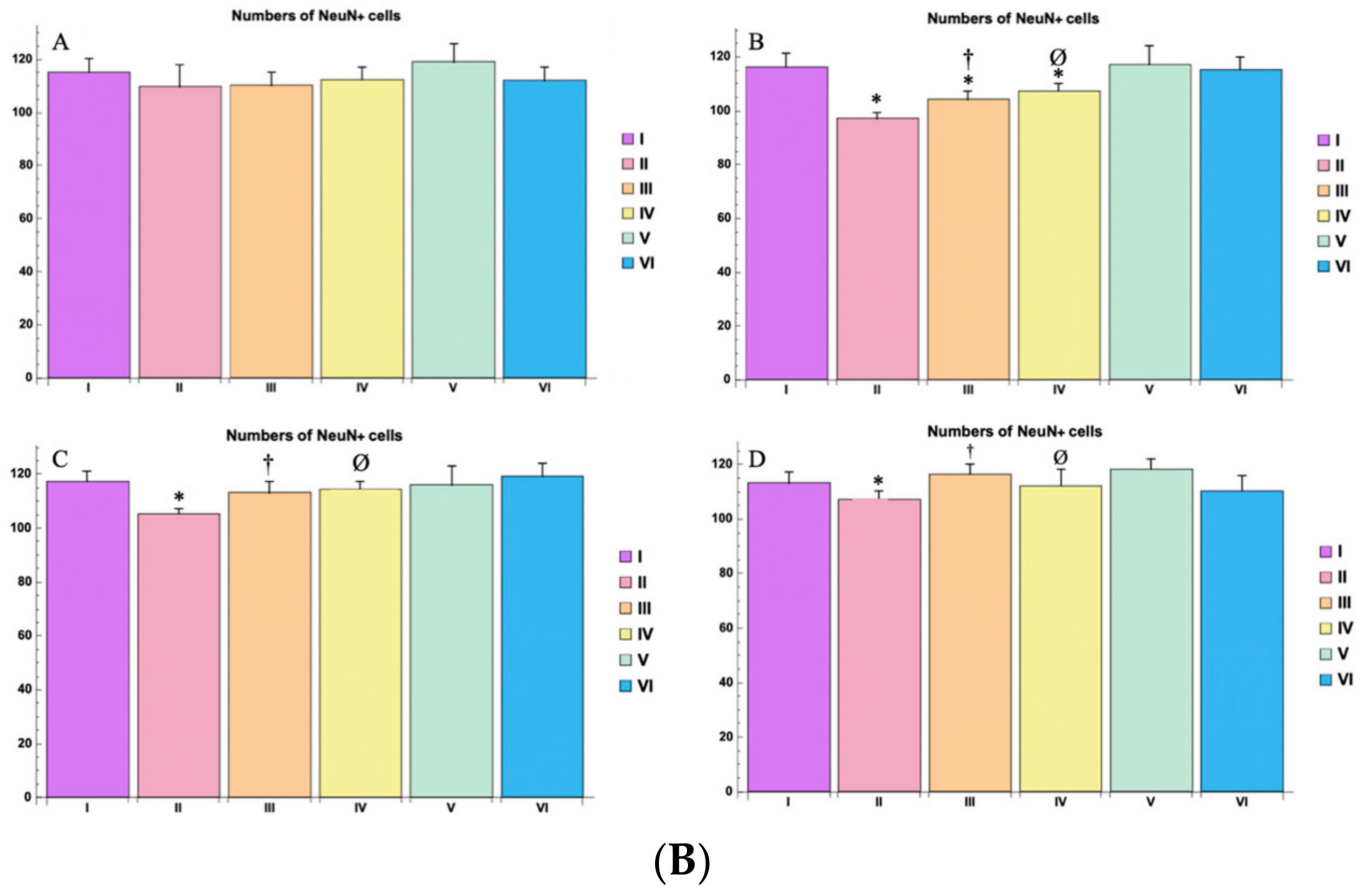
(**A**). Morphological picture NeuN+ cells in the hippocampus(dentate gyrus) at different time points of the experiment for differentstudy groups, magn. X400. In the group receiving only PTZ, after 24hours the degree of optical density of NeuN staining decreased. Andagainst the background of Myricetin administration (comparison drugValproic acid) the optical density of NeuN staining was higher. (**B**). The numbers of NeuN-positive-cells of dentate gyrus (the hippocampus zone) at different time points, per mm^2^: A—3 h, B—1 day, C—3 days, D—5 days. Experimental groups are numbered according to the study design. Data are presented as mean ±SD. Statistically significant differences compared to the control group are indicated by—* (*p * < 0.05); statistically significant differences between III and II are indicated by † (*p * < 0.05); statistically significant differences between IV and II groups are indicated by ø (*p * < 0.05). Groups: I—intact; II—PTZ only; III—Myricetin + PTZ; IV—Valproic Acid + PTZ; V—Myricetin + NaCl; VI—Valproic Acid + NaCl.

PTZ-induced seizures caused a significant reduction in NeuN-positive cells in the hippocampus after 24 h (after PTZ-injection), indicating neuronal damage. NeuN-positive cells were distributed in the hippocampus proper (regions CA1 and CA3) and in the granular layer of the dentate gyrus. While the intensity of NeuN staining varied across different regions and time points, the CA3 region exhibited the weakest labeling of hippocampal neurons, characterized by light nuclei, which is indicative of neuronal loss (*p * < 0.05). A similar decrease in NeuN-positive cells was observed in the CA1 and CA4 regions, as well as in the dentate gyrus, thalamic and hypothalamic nuclei, and motor structures such as the caudate nucleus and substantia nigra (*p * < 0.05). On the 3rd and 5th day after PTZ administration, there was a trend toward an increase in NeuN-positive neurons in the hippocampus and other brain regions. However, the number of NeuN-positive cells did not return to baseline levels (Figure 14A,B).

In contrast, in the Myricetin + PTZ group (group III) and Valproic Acid + PTZ group (group IV), the number of NeuN-positive cells was close to control values at all follow-up periods. Improved neuronal survival with more pronounced NeuN staining was detected in areas CA1, CA3 and CA4, as well as in the dentate gyrus (*p* < 0.05) (Figure 14A,B). At 3 days after PTZ, the number of NeuN-positive cells in the Myricetin and Valproic Acid groups was comparable to the control group, indicating significant neuroprotection (*p >* 0.05). On the 5th day, NeuN staining in these groups remained at the control level, indicating a sustained neuroprotective effect (Figure 14A,B).

In the control groups receiving Myricetin or Valproic Acid in combination with NaCl 0.9% (groups V and VI), the number of NeuN-positive cells did not differ significantly from the baseline of the control group. This indicates that these drugs alone did not exert neurotoxic effects (Figure 14A,B).

**Figure 15:**
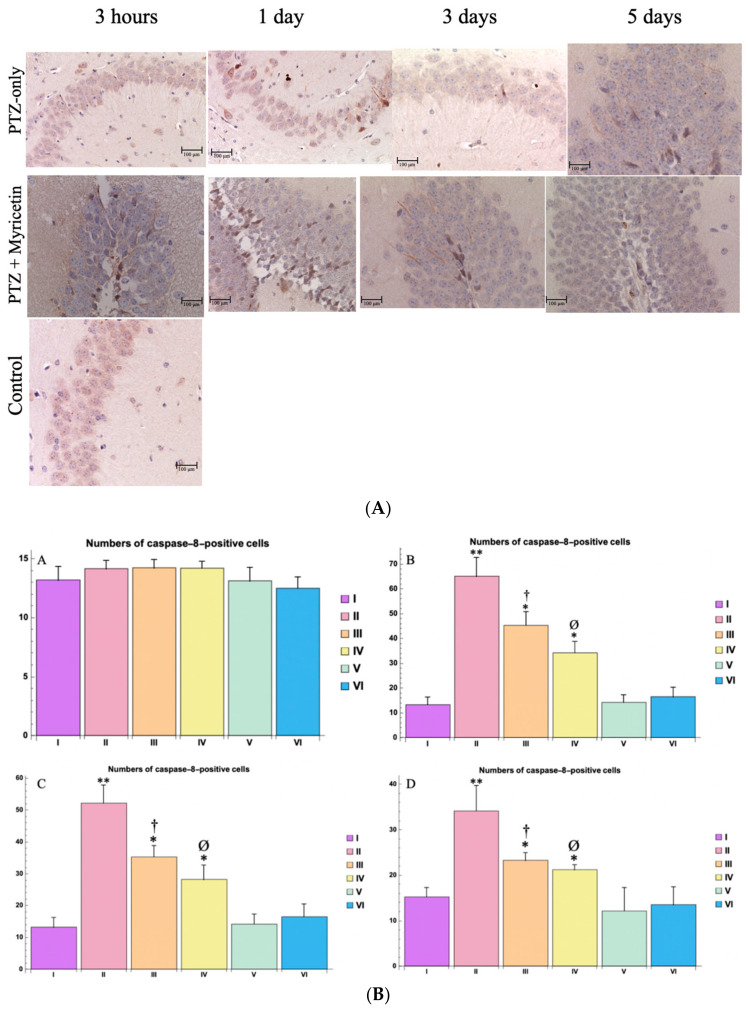
(**A**). Morphological picture of caspase-8^+^ cells in the hippocampus (dentate gyrus) at different time points of the experiment for different study groups, magn. ×400. Groups: I—intact; II—PTZ only; III—Myricetin + PTZ; IV—Valproic Acid + PTZ; V—Myricetin + NaCl; VI—Valproic Acid + NaCl. (**B**). The numbers of caspase-8-positive cells of dentate gyrus (the hippocampus zone) at different time points, per mm^2^: A—3 h, B—1 day, C—3 days, D—5 days. Experimental groups are numbered according to the study design. Data are presented as mean ±SD. Statistically significant differences compared to the control group are indicated by—* (*p * < 0.05), ** (*p * < 0.01); statistically significant differences between III and II are indicated by † (*p * < 0.01); statistically significant differences between IV and II groups are indicated by ø (*p * < 0.001). Groups: I—intact; II—PTZ only; III—Myricetin + PTZ; IV—Valproic Acid + PTZ; V—Myricetin + NaCl; VI—Valproic Acid + NaCl.

In the group receiving PTZ alone, significant immunohistochemical changes were observed in the hippocampus using antibodies to caspase-8. Diffuse staining of the cytoplasm was observed. Twenty-four hours after PTZ administration, there was a marked increase in the number of caspase-8-positive hippocampal neurons, indicating neuronal apoptosis against a background of a decrease in the number of NeuN-positive cells, indicating neuronal death in the CA1, CA3, and dentate gyrus regions (Figure 15A,B). On the 3rd and 5th day after PTZ administration, a tendency to decrease the number of Cascase-8-positive cells in the hippocampus structures and other brain regions was observed.

In the Myricetin + PTZ group, the neuroprotective effect of Myricetin was evident. The number of caspase-8-positive neurons in fields CA1, CA4, and the dentate gyrus was lower than in the PTZ only group. This indicates a decrease in neuronal apoptosis (Figure 15A,B).

The control groups receiving either Myricetin or Valproic Acid with NaCl 0.9% (groups V and VI) showed no significant differences in the number of cells with caspase-8 compared with the control group, indicating that these drugs alone did not result in neuronal damage or loss (Figure 15A,B).

Chemical bond damage to neurons induced by PTZ, accompanied by metabolic changes in enzyme and metabolite concentrations (MDA, SOD, and GSH) responsible for oxidative stress, as well as inflammatory markers (IL-1β, IL-6, and TNF-α), leads to a cumulative effect. This metabolic imbalance results in histological and immunohistochemical changes in neurons.

**Figure 16:**
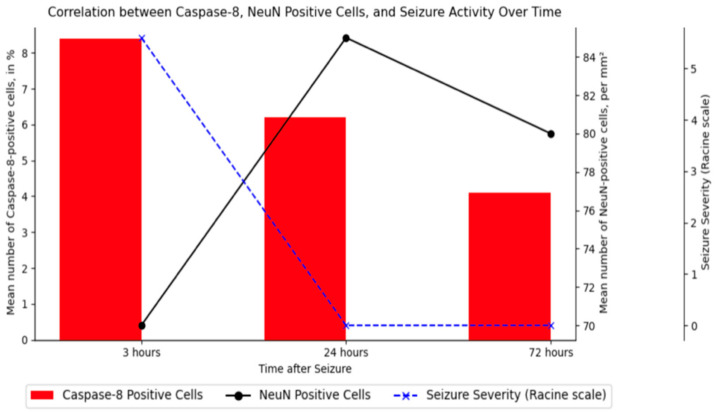
Correlation between caspase-8-positive cells, NeuN-positive cells, and seizure activity in the hippocampus. The graph illustrates the percentage of caspase-8-positive cells (indicating neuronal apoptosis), the number of NeuN-positive cells (indicating neuronal survival), and the severity of seizures on the Racine scale over time after PTZ-induced seizures. Data are presented for three time points: 3 h, 24 h, and 72 h post-seizure. The results show a decrease in caspase-8-positive cells, an increase followed by a decrease in NeuN-positive cells, and seizure severity only initially after PTZ administration.

In the PTZ-induced model, the surviving animals (*n* = 40) showed a significant increase in the number of damaged neurons (“dark”, caspase-8 positive) compared to control values in almost all structures. The most significant increase in the number of damaged neurons was found not only in the hippocampus but also in several limbic areas (especially posterior areas), thalamic and hypothalamic nuclei, and motor structures such as the caudate nucleus and substantia nigra. The level of NeuN-positive neurons in the hippocampus was inversely proportional to the level of caspase-8-positive cells (Figure 16).

The detected morphological and immunohistochemical (NeuN and caspase 8) changes in the neuronal composition of various brain regions, primarily the hippocampus, correlated with the intensity of behavioral responses (Figure 16).

According to the obtained biochemical data, the administration of Myricetin, like Valproic Acid, exerts an antioxidant effect on neurons, leading to metabolic and morphofunctional balance. Moderate increases in damaged neurons were recorded in the parietal cortex (*n* = 28), hippocampus, and dentate gyrus (*n* = 40) in the Myricetin and Valproic Acid administration groups. A similar pattern was observed in sensory areas and limbic structures, such as the mammillary body and cerebellar nuclei.

## 5. Discussions

The present study investigates the mechanisms of cell death in a PTZ-induced seizure model in mice by assessing neuronal markers such as NeuN and caspase-8 expression. Our findings represent the first exploration of Myricetin’s effects within this specific experimental context.

Traditionally, models like kindling are used to study epileptogenesis, cognitive and behavioral changes associated with epilepsy, and to evaluate new antiepileptic drugs [20,26]. However, we propose a novel, accelerated model of acute seizures induced by pentylenetetrazole (PTZ), validated through both pilot studies and our current experiment. Unlike the kindling model, which requires gradual, repetitive stimulation over an extended period, our approach focuses on the rapid induction of systemic seizures using subconvulsive doses of PTZ. This allows for the study of acute seizures and immediate treatment effects in a shorter time frame, offering a new perspective on the onset and progression of epileptic activity.

PTZ was chosen due to its well-documented high epileptogenic activity and the reproducibility of its effects via intraperitoneal administration [25,27]. While PTZ has been extensively used in kindling-like studies, our work is the first to describe the dynamic immunohistochemical changes in the hippocampus following an acute PTZ-induced seizure, for example, caspase-8 [5].

Our behavioral assessments revealed significant cognitive and motor impairments in the PTZ-only group, consistent with the hippocampus-dependent nature of the tasks employed [28]. These impairments, expected given the hippocampus’s vulnerability to damage, were accompanied by an astroglial response in the hippocampus without significant neuronal loss, aligning with previous research [29,30,31,32]. This test, along with others selected for their relevance to hippocampal function [33,34], revealed statistically significant differences primarily at the 3 h mark post-seizure. These results should be interpreted with caution, considering the neurotransmitter imbalances, the recovery time for basal levels, and energy deficits in affected animals. The rapid hippocampal neuron damage observed in this study is consistent with previous reports of long-lasting neuronal death or aberrant mossy fiber sprouting following severe ictal events [35,36].

To confirm our hypothesis regarding the key role of apoptotic mechanisms in neuronal damage, we assessed the expression of initiator caspase-8. The involvement of caspase-8 in triggering apoptosis within hippocampal structures following PTZ-induced seizures remains poorly understood.

Our immunohistochemical analysis revealed a significant decrease in NeuN-positive hippocampal neurons 24 h post-seizure, correlating with the morphological damage induced by systemic PTZ, as assessed by the Racine scale [37,38].

Our research is among the first that highlights the potential antiepileptic effects of Myricetin, a flavonoid recognized for its broad bioactive properties, including antioxidative, anticancer, nephroprotective, and anti-inflammatory effects [39,40,41]. In contrast is Valproic Acid, a well-established antiepileptic drug [33,42,43]. Limitations of using Valproic Acid underscore the need for alternative, less toxic therapeutic options. As expected, our behavioral assessments revealed significant cognitive and motor impairments in the PTZ-only group, consistent with the known effects of PTZ-induced seizures [44].

The astroglial response observed in the hippocampus of affected animals was notable but did not coincide with neuronal loss, aligning with findings from similar models [30,31,34,45]. These findings should be interpreted cautiously, considering factors such as neurotransmitter imbalance, recovery time for basal levels, and energy deficits in the animals. The rapid hippocampal neuron damage observed may explain the decreased function, which is consistent with previous reports of long-lasting neuronal death or aberrant mossy fiber sprouting following more severe ictal events, such as status epilepticus or kindling [46,47,48].

Immunohistochemical analysis revealed a decrease in NeuN-positive hippocampal neurons 24 h post-seizure, reflecting the morphological effects of systemic PTZ-induced seizures, as assessed by the Racine scale [37,38]. These results are consistent with similar studies on the morphological characterization of the hippocampus in epilepsy models [36].

Oxidative stress is a well-documented contributor to neuronal damage and seizure activity in epilepsy. The results of the study on markers of oxidative stress (MDA, SOD, and GSH) indicate that Myricetin and Valproic Acid effectively mitigate PTZ-induced oxidative stress.

The key mechanism by which Myricetin (3,3′,4′,4,5,5′,7-hexahydroxyflavone) suppresses oxidative stress involves the reduction in reactive oxygen species (ROS) through two main pathways: the neutralization of free radicals (O2•-, •OH, and ROO•) due to the presence of multiple hydroxyl groups in the compound’s chemical structure and the inhibition of pro-oxidant enzymes (xanthine oxidase, lipoxygenases, and NADPH oxidases). Additionally, Myricetin, like other flavonoids, can activate genes encoding antioxidant enzymes such as SOD, MDA, GSH, CAT, and GPx through the transcription factor Nrf2. The modulation of oxidative stress balance can also occur via signaling pathways (PI3K/Akt, MAPK, and NF-κB) influenced by Myricetin.

Myricetin demonstrated antioxidant and neuroprotective effects, resulting in significant improvements in cognitive and motor function compared to the group receiving PTZ alone in behavioral tests. The Valproic Acid administration group demonstrated similar results.

This antioxidant effect probably accounts for their neuroprotective properties in epilepsy, which provides a basis for further investigation of their therapeutic potential. Our findings are consistent with previous studies demonstrating the antioxidative and neuroprotective effects of similar compounds in models of epilepsy and other neurological disorders [49]. These results emphasize the potential of Myricetin and Valproic Acid as effective therapeutic agents in the treatment of epilepsy, which requires further investigation of their long-term effects and mechanisms of action.

Inflammation is a systemic factor in epileptogenesis, making neuroinflammatory markers a reliable predictor of epilepsy [50,51,52]. The present study aimed to assess the impact of PTZ-induced seizures and subsequent treatment with Myricetin and Valproic Acid on proinflammatory cytokines IL-1β, IL-6, and TNF-α in the serum of experimental animals.

The serum levels of IL-1β, IL-6, and TNF-α were significantly increased in the group receiving a single PTZ compared to the control group, emphasizing the role of inflammatory factors in neuronal damage and seizure activity. These findings are consistent with previous studies in other models of epilepsy, where the increase in cytokines was more pronounced compared to our work, where the acute phase was analyzed [53].

The most pronounced anti-inflammatory effects were observed 1 day post-PTZ administration, indicating the rapid onset of action for both Myricetin and Valproic Acid. At 3 h post-PTZ, cytokine levels (IL-1β, IL-6, and TNF-α) showed a moderate increase, marking the early inflammatory response. By 3 days post-PTZ, cytokine levels in the Myricetin and Valproic Acid groups had significantly decreased, approaching baseline, suggesting sustained anti-inflammatory effects. By 5 days post-PTZ, cytokine levels normalized across all groups, indicating a full resolution of inflammation. These findings suggest that both Myricetin and Valproic Acid effectively reduce PTZ-induced inflammation, likely contributing to their neuroprotective effects in epilepsy by mitigating neuronal damage and improving seizure outcomes.

Myricetin significantly reduced IL-1β, IL-6, and TNF-α levels, underscoring its strong anti-inflammatory properties. Similarly, Valproic Acid demonstrated a significant reduction in these cytokines, reinforcing its role in reducing inflammation. These results are consistent with previous research showing the anti-inflammatory effects of Myricetin and Valproic Acid in other neurological disorder models [54,55].

Additionally, morphological analysis using Nissl staining, along with NeuN and caspase-8 immunohistochemistry, provided evidence of the neuroprotective effects of Myricetin and Valproic Acid in the context of PTZ-induced seizures.

Nissl staining revealed a marked reduction in neuronal damage in the Myricetin-treated group compared to the PTZ-only group. Histopathological analysis showed that in the hippocampus, particularly in CA1, CA3, CA4, and the dentate gyrus, a higher density of viable neurons was maintained, and the number of “dark” neurons, characterized by pyknosis and hyperchromaticity, was significantly reduced. These findings suggest that Myricetin effectively mitigates PTZ-induced neuronal damage by providing a protective effect.

NeuN staining further confirmed these results. In the Myricetin + PTZ and Valproic Acid + PTZ groups, the number of NeuN-positive cells was significantly higher than in the PTZ-only group at all time points. NeuN-positive cells, indicative of neuronal integrity, were preserved in similar brain regions. By days 3 and 5 post-PTZ, the number of NeuN-positive cells in these groups was comparable to the control group, indicating significant neuroprotection and suggesting that both Myricetin and Valproic Acid contribute to the maintenance of neuronal survival and function.

To further investigate the molecular mechanisms of cell death in acute generalized seizures, we examined the role of the caspase cascade, focusing on caspase-8, which initiates apoptosis. This study is the first to use immunohistochemistry to explore caspase-8 activation in a model of acute epilepsy. Previous studies have identified caspase-8 as a critical initiator of apoptosis in hippocampal neuronal damage [56], with evidence suggesting that inhibiting caspase-8 increases the survival of neurons [57]. Our findings indicate that neuronal death in the hippocampus induced by PTZ is accompanied by the expression of caspase-8, a key marker of the cell death process. The distribution of damaged neurons may vary based on the brain region and type of epilepsy, likely due to the activation of specific signaling pathways involved in chemical and metabolic processes.

Our results align with previous studies that reported increased caspase-8 expression following systemic PTZ-induced seizures in mice [58]. However, it is important to note that these studies used immunoblotting, a non-quantitative method, whereas our study presents the first quantitative analysis of activated caspase-8 post-PTZ-induced seizure. The work of R. Meller et al. was particularly significant, as it demonstrated that caspase-8 activation leads to neuronal death during PTZ-induced seizures in the cell culture [57,58].

Immunohistochemical study for caspase-8 antibodies provided insight into apoptotic processes in the hippocampus. Given the defined role of an initiator of the extrinsic pathway of apoptosis, the elevation of caspase-8 levels in this work suggests with a high degree of probability that PTZ-induced activity arising from a single administration of a high dose of PTZ contributes to the initiation of the apoptotic pathway leading to cell death. A decrease in the number of caspase-8-positive cells was observed in the Myricetin + PTZ and Valproic Acid + PTZ groups compared to the group receiving PTZ only, indicating a reduction in apoptosis. The reduction in caspase-8 coincides with the observed preservation of NeuN-positive cells, reinforcing the neuroprotective role of these compounds. Additional work is required (including in another animal model) to determine the apoptotic pathway that leads to neuronal death after a single or after several repeated seizures, especially in the thalamus, fimbriae, amygdala, etc.

Summarizing the obtained data, systemic PTZ-induced seizures in mice reliably cause impaired neuronal function 24 h after the seizure, which normalizes by the 5th day post-experiment. The neuronal loss likely occurs through the extrinsic apoptosis pathway mediated by caspase-8 activation. The greatest abnormalities in behavioral tests were recorded 3 h after the seizure, indicating a critical period that requires further study. Additionally, the significant increase in pro-inflammatory cytokines (IL-1β, IL-6, and TNF-α) 24 h post-PTZ suggests a robust inflammatory response, which is mitigated by Myricetin and Valproic Acid. These treatments also demonstrated a pronounced antioxidant effect, as evidenced by the reduction in oxidative stress markers (MDA) and the restoration of antioxidant enzymes (SOD and GSH). The immunohistochemical findings, including NeuN and caspase-8 staining, reinforce the protective effects of Myricetin and Valproic Acid against PTZ-induced neuronal apoptosis. The improved outcomes in behavioral tests further substantiate their neuroprotective and therapeutic potential.

Thus, the results indicate that modeling systemic PTZ-induced seizures is a suitable model for studying the mechanisms of convulsive neuronal death and apoptosis. The findings highlight the promise of Myricetin as a therapeutic agent, warranting further investigation into its long-term benefits and mechanisms of action in epilepsy management.

## 6. Conclusions

PTZ-induced seizures result in significant neuronal damage and oxidative stress in the hippocampus, decreased NeuN-positive cells, and increased caspase-8 activity.

Myricetin administration showed promising neuroprotective effects. It significantly reduced oxidative stress markers, including MDA, and restored antioxidant enzyme activities (SOD and GSH). Myricetin also effectively attenuated the elevation of pro-inflammatory cytokines IL-1β, IL-6, and TNF-α. Behavioral assessments revealed that Myricetin improved cognitive and motor functions in PTZ-treated mice, with notable reductions in seizure severity and mortality rates. Morphology analyses supported these behavioral findings, with Nissl staining showing reduced neuronal damage and NeuN staining indicating better preservation of neuronal integrity in Myricetin-treated groups. Additionally, caspase-8 staining suggested a significant reduction in neuronal apoptosis.

The correlation between caspase 8 and Hey markers can act as predictors for immunohistochemical assessment and the prediction of the frequency or severity of attacks.

These results underscore the potential of Myricetin as a therapeutic agent in man-aging epilepsy through its antioxidative and anti-inflammatory actions, but with fewer side effects.

## Figures and Tables

**Table 1:** Modified Racine scale for Systemic PTZ-induced seizures in male CBA mice.

Seizure Type	Score	Behaviour
–	−1	normal
partial/focal seizures	0	whisker trembling
	1	sudden behavioural arrest
	2	facial jerking
generalized seizure	3	facial and neck jerking
	4	clonic seizure (sitting)
	5	clonic, tonic–clonic seizure
	6	clonic, tonic–clonic seizure and wild jumping
death	7	tonic extension, death

**Table 2:** Morris water maze test results. Time spent in target zone. Data are presented for Groups 2, 3, and 4 only.

Group		Time, s	*p*-Value
Baseline		39.64 ± 8.94	-
3 h	Myricetin + PTZ	38.14 ± 3.83	0.02
	Valproic Acid + PTZ	39.49 ± 3.11	
	PTZ	31.22 ± 2.85	
1 day	Myricetin + PTZ	38.33 ± 4.32	0.36
	Valproic Acid + PTZ	37.49 ± 5.11	
	PTZ	35.98 ± 5.41	
3 days	Myricetin + PTZ	35.03 ± 5.49	0.29
	Valproic Acid + PTZ	41.05 ± 3.27	
	PTZ	38.61 ± 3.59	
5 days	Myricetin + PTZ	39.17 ± 5.04	0.66
	Valproic Acid + PTZ	39.32 ± 3.27	
	PTZ	40.33 ± 4.54	

## Data Availability

Data are available upon request to the corresponding author.

## References

[1] do Canto A.M., Donatti A., Geraldis J.C., Godoi A.B., da Rosa D.C., Lopes-Cendes I. (2021). Neuroproteomics in Epilepsy: What Do We Know So Far?. Front. Mol. Neurosci..

[2] Milligan T.A. (2021). Epilepsy: A Clinical Overview. Am. J. Med..

[3] Thom M. (2014). Review: Hippocampal sclerosis in epilepsy: A neuropathology review. Neuropathol. Appl. Neurobiol..

[4] Blümcke I., Thom M., Aronica E., Armstrong D.D., Bartolomei F., Bernasconi A., Bernasconi N., Bien C.G., Cendes F., Coras R. (2013). International consensus classification of hippocampal sclerosis in temporal lobe epilepsy: A Task Force report from the ILAE Commission on Diagnostic Methods. Epilepsia.

[5] Nehlig A., Pereira de Vasconcelos A. (1996). The model of pentylenetetrazol-induced status epilepticus in the immature rat: Short- and long-term effects. Epilepsy Res..

[6] Alachkar A., Ojha S.K., Sadeq A., Adem A., Frank A., Stark H., Sadek B. (2020). Experimental Models for the Discovery of Novel Anticonvulsant Drugs: Focus on Pentylenetetrazole-Induced Seizures and Associated Memory Deficits. Curr. Pharm. Des..

[7] Thapliyal S., Garg N., Joshi R., Chakrabarti A., Medhi B. (2023). Pentylenetetrazole Induced Kindling Model of Refractory Epilepsy: A Proof-of-concept Study to Explore Dose and Time Range of Phenobarbital in Rats. Basic. Clin. Neurosci..

[8] Wolf H.K., Buslei R., Schmidt-Kastner R., Schmidt-Kastner P.K., Pietsch T., Wiestler O.D., Blümcke I. (1996). NeuN: A useful neuronal marker for diagnostic histopathology. J. Histochem. Cytochem..

[9] Mjønes P., Sagatun L., Nordrum I.S., Waldum H.L. (2017). Neuron-Specific Enolase as an Immunohistochemical Marker Is Better than Its Reputation. J. Histochem. Cytochem..

[10] Chang L.R., Liu J.P., Song Y.Z., Lu T., Lu G., Wu Y. (2011). Expression of caspase-8 and caspase-9 in rat hippocampus during postnatal development. Microsc. Res. Tech..

[11] Kulikov A.A., Dorofeeva N.A., Naumova A.A., Harbachova E.L., Glazova M.V., Chernigovskaya E.V. (2020). Impaired postnatal development of the hippocampus of Krushinsky-Molodkina rats genetically prone to audiogenic seizures. Epilepsy Behav..

[12] Basaranlar G., Derin N., Kencebay Manas C., Tanriover G., Aslan M. (2019). The effects of sulfite on cPLA2, caspase-3, oxidative stress and locomotor activity in rats. Food Chem. Toxicol..

[13] Narkilahti S., Pitkänen A. (2005). Caspase 6 expression in the rat hippocampus during epileptogenesis and epilepsy. Neuroscience.

[14] Tzeng T.T., Tsay H.J., Chang L., Hsu C.-L., Lai T.-H., Huang F.-L., Shiao Y.-J. (2013). Caspase 3 involves in neuroplasticity, microglial activation and neurogenesis in the mice hippocampus after intracerebral injection of kainic acid. J. Biomed. Sci..

[15] Nguyen T.T.M., Gillet G., Popgeorgiev N. (2021). Caspases in the Developing Central Nervous System: Apoptosis and Beyond. Front. Cell Dev. Biol..

[16] Sharangpani A., Takanohashi A., Bell M.J. (2008). Caspase activation in fetal rat brain following experimental intrauterine inflammation. Brain Res..

[17] Henshall D.C., Bonislawski D.P., Skradski S.L., Lan J.Q., Meller R., Simon R.P. (2001). Cleavage of bid may amplify caspase-8-induced neuronal death following focally evoked limbic seizures. Neurobiol. Dis..

[18] Kim H.H., Kim D.H., Kim M.H., Oh M.H., Kim S.R., Park K.J., Lee M.W. (2013). Flavonoid constituents in the leaves of *Myrica rubra* sieb. et zucc. with anti-inflammatory activity. Arch. Pharm. Res..

[19] Shimosaki S., Tsurunaga Y., Itamura H., Nakamura M. (2011). Anti-allergic effect of the flavonoid myricitrin from *Myrica rubra* leaf extracts in vitro and in vivo. Nat. Prod. Res..

[20] Sun Z.Q., Meng F.H., Tu L.X., Sun L. (2019). Myricetin attenuates the severity of seizures and neuroapoptosis in pentylenetetrazole kindled mice by regulating the BDNF-TrkB signaling pathway and modulating matrix metalloproteinase-9 and GABAA. Exp. Ther. Med..

[21] Van Erum J., Van Dam D., De Deyn P.P. (2019). PTZ-induced seizures in mice require a revised Racine scale. Epilepsy Behav..

[22] Monville C., Torres E.M., Dunnett S.B. (2006). Comparison of incremental and accelerating protocols of the rotarod test for the assessment of motor deficits in the 6-OHDA model. J. Neurosci. Methods.

[23] Prut L., Belzung C. (2003). The open field as a paradigm to measure the effects of drugs on anxiety-like behaviors: A review. Eur. J. Pharmacol..

[24] Vorhees C.V., Williams M.T. (2006). Morris water maze: Procedures for assessing spatial and related forms of learning and memory. Nat. Protoc..

[25] Shimada T., Yamagata K. (2018). Pentylenetetrazole-Induced Kindling Mouse Model. J. Vis. Exp. Jove.

[26] Bertram E. (2007). The relevance of kindling for human epilepsy. Epilepsia.

[27] Löscher W. (2011). Critical review of current animal models of seizures and epilepsy used in the discovery and development of new antiepileptic drugs. Seizure.

[28] Lu Z., Teng Y., Wang L., Jiang Y., Li T., Chen S., Wang B., Li Y., Yang J., Wu X. (2022). Abnormalities of hippocampus and frontal lobes in heart failure patients and animal models with cognitive impairment or depression: A systematic review. PLoS ONE.

[29] Díaz F., Aguilar F., Wellmann M., Martorell A., González-Arancibia C., Chacana-Véliz L., Negrón-Oyarzo I., Chávez A.E., Fuenzalida M., Nualart F. (2023). Enhanced Astrocyte Activity and Excitatory Synaptic Function in the Hippocampus of Pentylenetetrazole Kindling Model of Epilepsy. Int. J. Mol. Sci..

[30] Pavlova T., Stepanichev M., Gulyaeva N. (2006). Pentylenetetrazole kindling induces neuronal cyclin B1 expression in rat hippocampus. Neurosci. Lett..

[31] Nizinska K., Szydlowska K., Vouros A., Kiryk A., Stepniak A., Vasilaki E., Lukasiuk K. (2021). Behavioral characteristics as potential biomarkers of the development and phenotype of epilepsy in a rat model of temporal lobe epilepsy. Sci. Rep..

[32] Pilly P.K., Grossberg S. (2012). How do spatial learning and memory occur in the brain? Coordinated learning of entorhinal grid cells and hippocampal place cells. J. Cogn. Neurosci..

[33] Burman D.D. (2019). Hippocampal connectivity with sensorimotor cortex during volitional finger movements: Laterality and relationship to motor learning. PLoS ONE.

[34] Sullens D.G., Nguyen P., Gilley K., Wiffler M.B., Sekeres M.J. (2023). Hippocampal motor memory network reorganization depends on familiarity, not time. Learn. Mem..

[35] Zhang L., Guo Y., Hu H., Wang J., Liu Z., Gao F. (2015). FDG-PET and NeuN-GFAP immunohistochemistry of hippocampus at different phases of the pilocarpine model of temporal lobe epilepsy. Int. J. Med. Sci..

[36] Boulle F., Massart R., Stragier E., Païzanis E., Zaidan L., Marday S., Gabriel C., Mocaer E., Mongeau R., Lanfumey L. (2014). Hippocampal and behavioral dysfunctions in a mouse model of environmental stress: Normalization by agomelatine. Transl. Psychiatry.

[37] Lopim G.M., Vannucci Campos D., Gomes da Silva S., de Almeida A.A., Lent R., Cavalheiro E.A., Arida R.M. (2016). Relationship between seizure frequency and number of neuronal and non-neuronal cells in the hippocampus throughout the life of rats with epilepsy. Brain Res..

[38] Domitrović R., Rashed K., Cvijanović O., Vladimir-Knežević S., Škoda M., Višnić A. (2015). Myricitrin exhibits antioxidant, anti-inflammatory and antifibrotic activity in carbon tetrachloride-intoxicated mice. Chem. Biol. Interact..

[39] Bai Y., Liu X., Chen Q., Chen T., Jiang N., Guo Z. (2021). Myricetin ameliorates ox-LDL-induced HUVECs apoptosis and inflammation via lncRNA GAS5 upregulating the expression of miR-29a-3p. Sci. Rep..

[40] Hassan S.M., Khalaf M.M., Sadek S.A., Abo-Youssef A.M. (2017). Protective effects of apigenin and myricetin against cisplatin-induced nephrotoxicity in mice. Pharm. Biol..

[41] Tomson T., Battino D., Perucca E. (2016). Valproic acid after five decades of use in epilepsy: Time to reconsider the indications of a time-honoured drug. Lancet Neurol..

[42] Khodayar M.J., Kalantari H., Khorsandi L., Ahangar N., Samimi A., Alidadi H. (2021). Taurine attenuates Valproic acid-induced hepatotoxicity via modulation of RIPK1/RIPK3/MLKL-mediated necroptosis signaling in mice. Mol. Biol. Rep..

[43] Caiment F., Wolters J., Smit E., Schrooders Y., Kleinjans J., van den Beucken T. (2020). Valproic acid promotes mitochondrial dysfunction in primary human hepatocytes in vitro; impact of C/EBPα-controlled gene expression. Arch. Toxicol..

[44] de Melo A.D., Freire V.A.F., Diogo Í.L., Santos H.L., Barbosa L.A., de Carvalho L.E.D. (2023). Antioxidant Therapy Reduces Oxidative Stress, Restores Na,K-ATPase Function and Induces Neuroprotection in Rodent Models of Seizure and Epilepsy: A Systematic Review and Meta-Analysis. Antioxidants.

[45] Yang N., Guan Q.W., Chen F.H., Xia Q.-X., Yin X.-X., Zhou H.-H., Mao X.-Y. (2020). Antioxidants Targeting Mitochondrial Oxidative Stress: Promising Neuroprotectants for Epilepsy. Oxidative Med. Cell. Longev..

[46] Alvim M.K.M., Morita-Sherman M.E., Yasuda C.L., Rocha N.P., Vieira E.L., Pimentel-Silva L.R., Nogueira M.H., Barbosa R., Watanabe N., Coan A.C. (2021). Inflammatory and neurotrophic factor plasma levels are related to epilepsy independently of etiology. Epilepsia.

[47] Terrone G., Balosso S., Pauletti A., Ravizza T., Vezzani A. (2020). Inflammation and reactive oxygen species as disease modifiers in epilepsy. Neuropharmacology.

[48] de Vries E.E., van den Munckhof B., Braun K.P., van Royen-Kerkhof A., de Jager W., Jansen F.E. (2016). Inflammatory mediators in human epilepsy: A systematic review and meta-analysis. Neurosci. Biobehav. Rev..

[49] Kamaşak T., Dilber B., Yaman S.Ö., Durgut B.D., Kurt T., Çoban E., Arslan E.A., Şahin S., Karahan S.C., Cansu A. (2020). HMGB-1, TLR4, IL-1R1, TNF-α, and IL-1β: Novel epilepsy markers?. Epileptic Disord..

[50] Uludag I.F., Duksal T., Tiftikcioglu B.I., Zorlu Y., Ozkaya F., Kirkali G. (2015). IL-1β, IL-6 and IL1Ra levels in temporal lobe epilepsy. Seizure.

[51] Guo A., Li J., Luo L., Chen C., Lu Q., Ke J., Feng X. (2021). Valproic acid mitigates spinal nerve ligation-induced neuropathic pain in rats by modulating microglial function and inhibiting neuroinflammatory response. Int. Immunopharmacol..

[52] Boriero D., Carcereri de Prati A., Antonini L., Ragno R., Sohji K., Mariotto S., Butturini E. (2021). The anti-STAT1 polyphenol myricetin inhibits M1 microglia activation and counteracts neuronal death. FEBS J..

[53] Wu S., Yue Y., Peng A., Zhang L., Xiang J., Cao X., Ding H., Yin S. (2016). Myricetin ameliorates brain injury and neurological deficits via Nrf2 activation after experimental stroke in middle-aged rats. Food Funct..

[54] Cao G., Luo Y., Nagayama T., Pei W., Stetler R.A., Graham S.H., Chen J. (2002). Cloning and characterization of rat caspase-9: Implications for a role in mediating caspase-3 activation and hippocampal cell death after transient cerebral ischemia. J. Cereb. Blood Flow. Metab..

[55] Krajewska M., You Z., Rong J., Kress C., Huang X., Yang J., Kyoda T., Leyva R., Banares S., Hu Y. (2011). Neuronal deletion of caspase 8 protects against brain injury in mouse models of controlled cortical impact and kainic acid-induced excitotoxicity. PLoS ONE.

[56] Wen S., Wang L., Zhang W., Xu M., Song R., Zou H., Gu J., Bian J., Yuan Y., Liu Z. (2021). Induction of mitochondrial apoptosis pathway mediated through caspase-8 and c-Jun N-terminal kinase by cadmium-activated Fas in rat cortical neurons. Metallomics.

[57] Ahmadpour S., Behrad A., Vega I.F. (2019). Dark Neurons: A protective mechanism or a mode of death. J. Med. Histol..

[58] Meller R., Clayton C., Torrey D.J., Schindler C., Lan J., Cameron J., Chu X., Xiong Z., Simon R., Henshall D. (2006). Activation of the caspase 8 pathway mediates seizure-induced cell death in cultured hippocampal neurons. Epilepsy Res..

